# An aromatic noble-gas hydride: C_6_H_5_CCXeH

**DOI:** 10.1038/s41598-017-02869-9

**Published:** 2017-06-09

**Authors:** Luís Duarte, Leonid Khriachtchev

**Affiliations:** Department of Chemistry, University of Helsinki, P.O. Box 55, FI-00014 Helsinki, Finland

## Abstract

We report on the aromatic noble-gas hydride, C_6_H_5_CCXeH, identified in a xenon matrix using infrared spectroscopy and extensive quantum chemical calculations. This molecule is prepared by 250-nm photolysis of phenylacetylene (C_6_H_5_CCH) isolated in a xenon matrix and subsequent thermal mobilization of hydrogen atoms at about 40 K. The characteristic H–Xe stretching mode of C_6_H_5_CCXeH is observed at about 1500 cm^−1^, and a number of other fundamentals also appear in the experimental spectra. The assignment is supported by deuteration experiments providing predictable shifts of the vibrational frequencies. The experimental and calculated spectra are in a good agreement. C_6_H_5_CCXeH is computationally lower in energy than the C_6_H_5_CC + Xe + H fragments by about 0.60 eV at the M06-2X/aug-cc-pVTZ-PP level of theory, which allows its formation at low temperatures. C_6_H_5_CCXeH is the first aromatic noble-gas hydride and the first halogen-free aromatic noble-gas compound.

## Introduction

The discovery of the first xenon compound by Neil Bartlett started experimental noble-gas chemistry^1^. A number of molecules with krypton, xenon, and radon atoms were reported quickly after Bartlett’s breakthrough^2–4^. A large number of noble-gas compounds have been identified to date^5^. In particular, a few aromatic noble-gas molecules containing fluorine and chlorine have been prepared. For example, Frohn *et al*. synthetized C_6_F_5_XeF and C_6_F_5_XeCl^6–8^; however, all aromatic noble-gas compounds contain halogens. A very recent success in noble-gas chemistry has been the identification of Na_2_He at very high pressures^9^.

Another important development in this field was the discovery of noble-gas hydrides HNgY (Ng = a noble-gas atom; Y = an electronegative fragment) in cryogenic matrices^10^. HNgY molecules are characterized by a charge-transfer character with the positively-charged HNg group and the negatively-charged Y fragment^11^. The H–Ng bond is mainly covalent and the Ng–Y bond is mainly ionic. All experimentally prepared molecules are computationally lower in energy than the H + Ng + Y asymptote (three-body dissociation channel), which ensures their stability at low temperatures. On the other hand, HNgY molecules are metastable with respect to the global minimum Ng + HY; however, they are protected from decomposition by a relatively high bending barrier. The HNgY molecules are typically prepared by UV photolysis (or radiolysis) of an HY/Ng matrix and thermal mobilization of produced H atoms and identified by infrared spectroscopy benefiting from strong intensity of the H–Xe stretching mode. By now, about 30 HNgY molecules have been reported^5, 11^. This approach allowed to identify the only experimentally known neutral argon molecule (HArF)^12, 13^. Other remarkable members of this family are the halogen-free organo-xenon and organo-krypton compounds (HXeCCH, HKrCCH, HXeCCXeH, etc.)^14–17^.

Two aromatic noble-gas hydrides resulting from the insertion of xenon into benzene and phenol were computationally predicted more than ten years ago^18^. However, the experimental efforts aiming at their preparation have not been successful, presumably because of the lack of energetic stability^19, 20^. Thus, other candidates should be found to prepare this challenging type of molecules. Phenylacetylene (C_6_H_5_CCH, PhAc) is a promising precursor for this task. Although the electronegativity of phenylethynyl radical (C_6_H_5_CC) is unknown, its electron affinity is predicted to be close to that of CCH^21^. One may expect that the electronegativities of these two radicals are also similar. In this situation, the reaction C_6_H_5_CC + Xe + H is realistic taking into account that the reaction of HCC + Xe + H leads to HCCXeH^14, 16^. However, the possibility to generate phenylethynyl radicals in a xenon matrix remains the critical point.

Here, we report on the identification of an aromatic noble-gas hydride, C_6_H_5_CCXeH, prepared by photolysis and annealing of xenon matrices doped with PhAc. The assignments are supported by experiments with deuterated PhAc and by extensive quantum-chemical calculations.

## Results and Discussion

### Computational results

**Figure 1:**
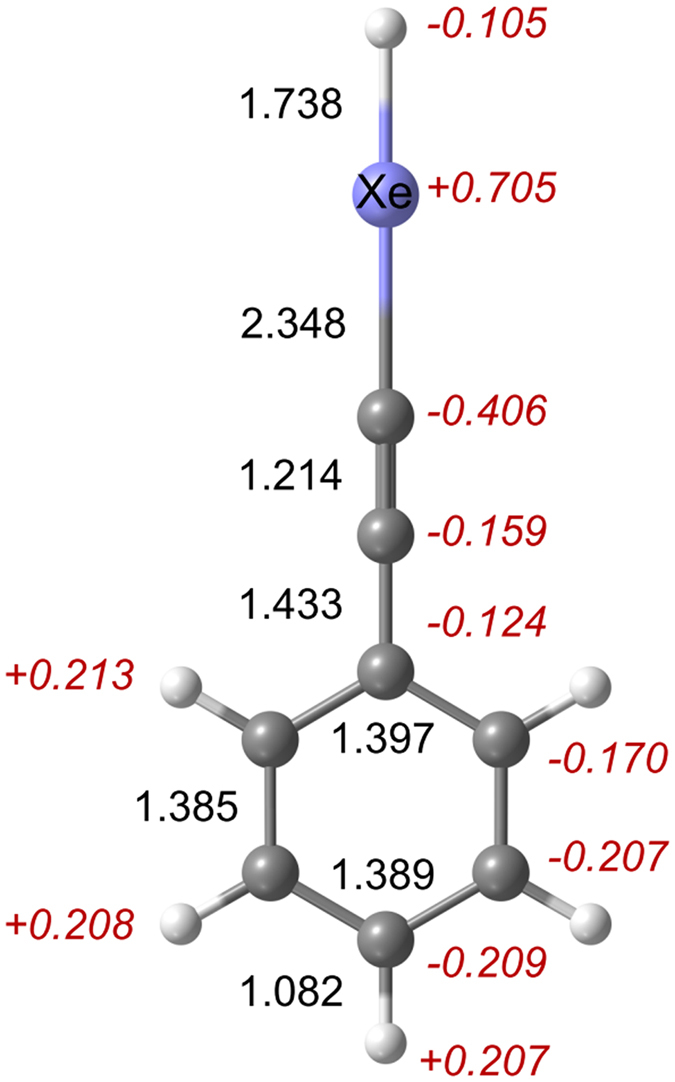
Equilibrium structure of C_6_H_5_CCXeH calculated at the M06-2X/aug-cc-pVTZ-PP level of theory. The bond lengths (normal font) are in angstroms and the NPA atomic charges (italics) are in elementary charges.

C_6_H_5_CCXeH is a true energy minimum and has *C*_2v_ symmetry. The equilibrium structure, bond lengths, and NPA atomic charges calculated at the M06-2X/aug-cc-pVTZ-PP level of theory are given in Fig. 1. The H–Xe and Xe–C bond distances are 1.738 and 2.348 Å. The charges on the Xe atom and the H atom bound to Xe are + 0.705 and −0.105 (in elementary charges). Most of the negative partial charge is located on C atoms of the C_6_H_5_CC group, particularly on the C atom bound to Xe (−0.406). The aromatic H atoms are positively charged. Compared to PhAc, the insertion of a xenon atom affects mainly the C≡C group: this bond elongates by 0.015 Å (other bonds change by ≤0.001 Å) and the charges of these C atoms become more negative by −0.136 and −0.199. The results obtained at different levels of theory are shown in Tables S1 and S2 in the Supplementary Information.

At the M06-2X level of theory, C_6_H_5_CCXeH is 0.60 eV lower in energy than the C_6_H_5_CC + Xe + H fragments (after ZPVE correction). It follows that the experimental annealing-induced formation of C_6_H_5_CCXeH is possible under matrix-isolation conditions. As other HNgY molecules, C_6_H_5_CCXeH is a metastable species with respect to the C_6_H_5_CCH + Xe global minimum (by 4.9 eV), but it is presumably protected from decomposition by a high bending barrier^11^. The compounds resulting from the insertion of Ar and Kr into PhAc are predicted to be higher in energy than the C_6_H_5_CC + Ng + H fragments and their formation is not expected in the experiment. To recall, all experimentally observed HNgY molecules are computationally below the H + Ng + Y energy assymptote^11, 22^. The energetics of the C_6_H_5_CCNgH molecules at different levels of theory is shown in Table S3 in the Supplementary Information.

The calculated harmonic frequencies and infrared intensities of C_6_H_5_CCXeH and C_6_D_5_CCXeD are listed in Table S4 in the Supplementary Information. The characteristic H–Xe stretching frequency is 1748.1 cm^−1^ with an intensity of 1586 km mol^−1^ at the M06-2X level. This frequency significantly decreases upon deuteration to 1242.1 cm^−1^ (732 km mol^−1^). Other absorptions with predicted non-negligible intensities (~90 km mol^−1^ *)* are at 1237.5, 796.0, and 525.9 cm^−1^ (1171.4, 744.6 and 514.9 cm^−1^ upon deuteration) and they correspond to the C–C≡ stretching and two CCC ring bending modes. The H–Xe–C in-plane and out-of-plane bending modes are at 661.1 cm^−1^ and 658.0 cm^−1^ (481.0 and 473.5 cm^−1^ upon deuteration) and they are predicted to have relatively weak intensities (~2–4 km mol^−1^). The Xe–C stretching and XeCC bending modes at 163.3 cm^−1^ and ~34 cm^−1^ (160.2 and ~33 cm^−1^ upon deuteration) are obviously out of our experimental range.

It is worth comparing the calculated properties of C_6_H_5_CCXeH and HXeCCH. For HXeCCH, the H–Xe and Xe–C bond distances (1.738 and 2.352 Å at the M06-2X level) are similar to those of C_6_H_5_CCXeH. The atomic charges in HXeCCH are −0.116 on H (bound to Xe), +0.719 on Xe, and −0.468 on C (bound to Xe) atoms. It is seen that the positive partial charge on the HXe group and the negative partial charge on the C atom bound to Xe are very similar for these two molecules. The calculated H–Xe and Xe–C stretching frequencies of HXeCCH are 1751.3 and 306.3 cm^−1^ (M06-2X), featuring the higher frequency of the Xe–C stretching mode than in C_6_H_5_CCXeH. At the same level of theory (M06-2X), HXeCCH is 0.70 eV lower in energy than the H + Xe + CCH fragments and about 4.9 eV higher in energy than the HCCH + Xe global minimum (after ZPVE correction). These values are similar to the ones (−1.5 and 4.5 eV) calculated at the MP2/LJ18/6-311 ++G(2d, 2p) level^18^.

We also calculated the properties of PhAc, PhAc-d_6_, and PhAc-d_1_ and a number of species that can appear upon photolysis of the precursors particularly the phenylethynyl (C_6_H_5_CC) and ethynylphenyl (C_6_H_4_CCH) radicals. The results are shown in Tables S5–S7 and Fig. S1 in the Supplementary Information. Nucleus-independent chemical shifts of PhAc and C_6_H_5_CCXeH were calculated at the M06-2X level. The aromaticity of these two molecules is found to be very similar (see Table S8 in the Supplementary Information).

### Experimental results and assignment

**Figure 2:**
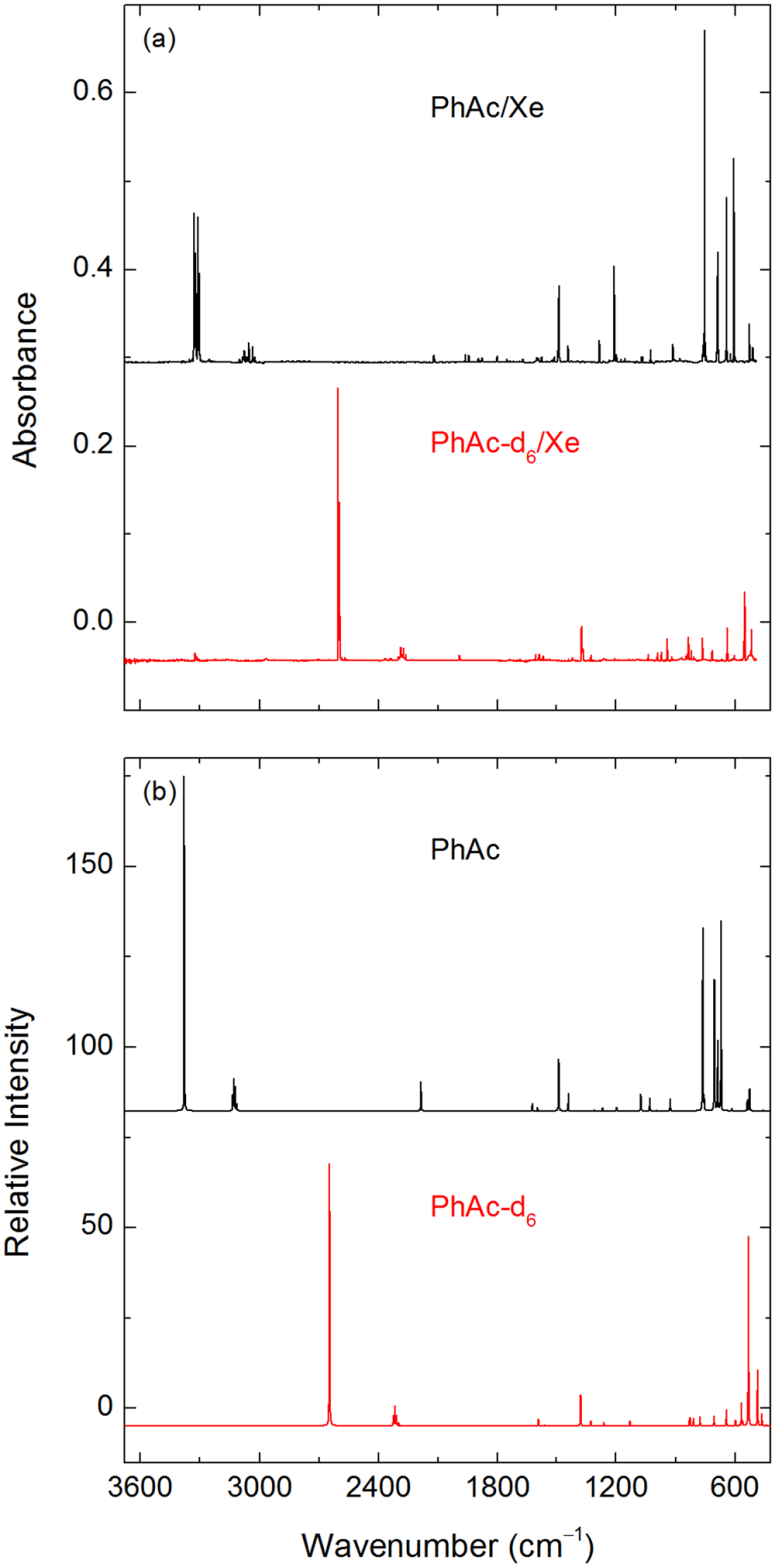
FTIR spectra of PhAc (upper trace) and PhAc-d_6_ (lower trace) in a xenon matrix (**a**). Calculated spectra at the M06-2X/aug-cc-pVTZ level of theory and scaled by a factor of 0.97 (**b**).

Figure 2 shows the experimental (xenon matrix) and calculated (M06-2X) spectra of PhAc and PhAc-d_6_. There is a good agreement between the experimental and calculated spectra (see also Table S5 in the Supplementary Information). Other levels of theory predict similar spectra. In the ≡C–H stretching region, PhAc has two intense multiplets centered at ca. 3325 and 3307 cm^−1^ in a xenon matrix (3332 and 3313 cm^−1^ in a krypton matrix; 3339 and 3323 cm^−1^ in an argon matrix). Other characteristic absorptions in a xenon matrix appear at 755, 688, 642, and 607 cm^−1^ (756, 688, 644, and 608 cm^−1^ in a krypton matrix; 756, 689, 647, and 610 cm^−1^ in an argon matrix), corresponding to the phenyl CH out-of-plane, ≡C–H in-plane, phenyl CH out-of-plane, and ≡C–H out-of-plane bending modes, respectively. These frequencies are in good agreement with the values previously reported for the ≡C–H stretching band (3323.2 and 3310.8/3309.2 cm^−1^) and the phenyl CH out-of-plane bending band (758.7 cm^−1^) in a nitrogen matrix^23, 24^. The splitting of the ≡C–H stretching band was explained by a Fermi resonance between the acetylenic ≡C–H stretching vibration and a combination of one quantum of the C≡C stretching vibration and two quanta of the C≡C–H out-of-plane bending vibration^25–27^. In the deuterated species (PhAc-d_6_), the Fermi resonance is suppressed due to the shift of the ≡C–D stretching mode to a lower energy. However, some splitting still occurs in the ≡C–D stretching region (2601.5/2595.0/2592.5 cm^−1^) probably due to different matrix sites. A similar situation occurs in a nitrogen matrix^23, 24^.

**Figure 3:**
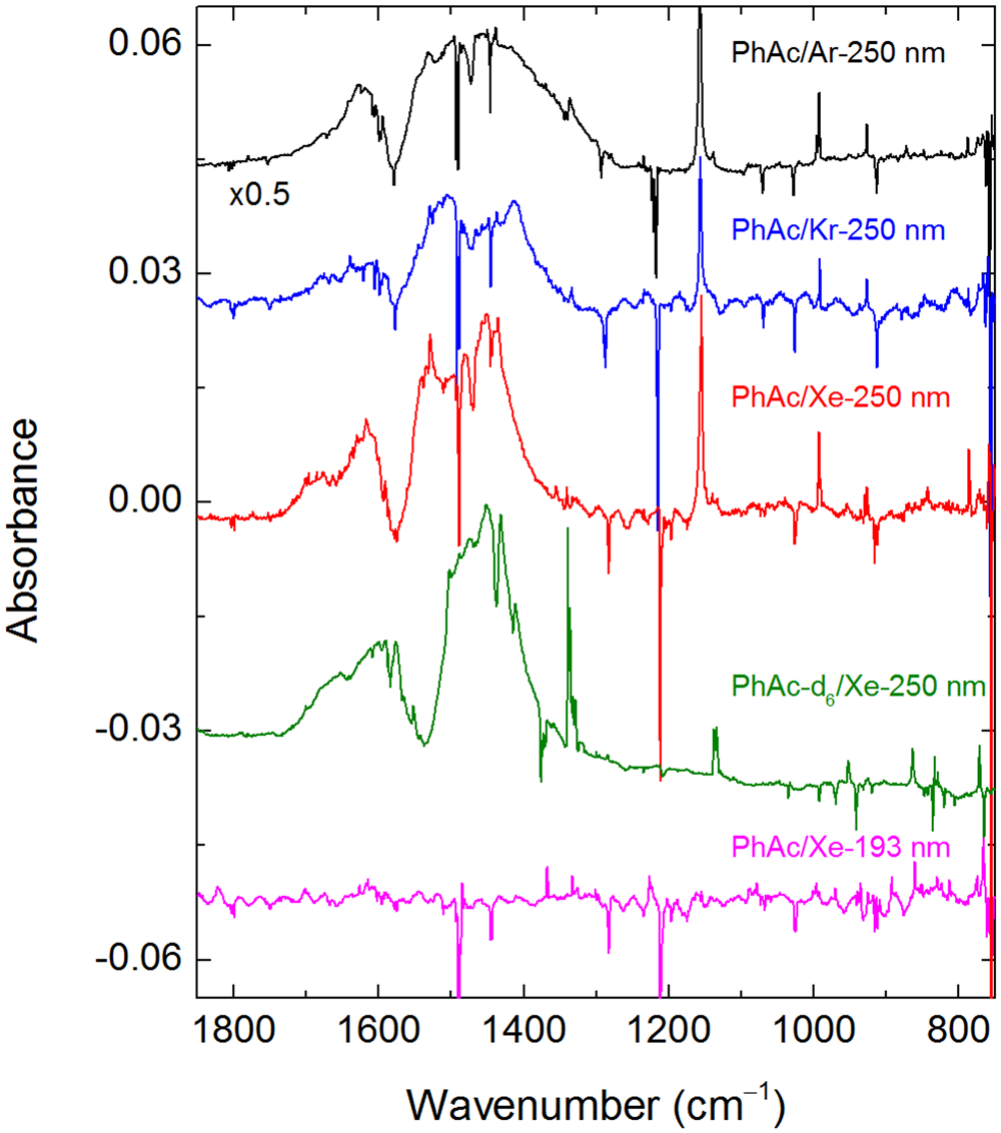
UV photolysis of PhAc in noble-gas matrices. The difference FTIR spectra show (from top to bottom) the results of photolysis of PhAc/Ng matrices (Ng = Ar, Kr, and Xe) at 250 nm, of a PhAc-d_6_/Xe matrix at 250 nm, and of a PhAc/Xe matrix at 193 nm. The negative bands originate from PhAc.

Upon 250-nm photolysis, matrix-isolated PhAc is consumed (typically by 20–30% after 3–7 × 10^4^ pulses) and a distinct set of bands appears in the spectrum (Fig. 3), assigned here to phenylethynyl radical. Two broad and structured absorptions are observed in the 2400 to 2230 cm^−1^ (medium intensity) and 1700 to 1300 cm^−1^ (strong intensity) regions. We connect these absorptions with the presence of low-energy electronic transitions that are also known for closely-related CCX radicals (X = H, F, Cl, and Br)^28–32^. The accurate assignment of vibronic transition is a very complicated task even for the three-atom radicals and probably impossible for phenylethynyl radical. On the other hand, the well-defined bands in the lower-energy region (below the broad bands) show a good agreement with the calculated vibrational transitions of phenylethynyl radical (Table 1)^33^. These bands correlate at different stages of experiment with each other and with the broad bands; thus, they belong to the same species. After photolysis, XeHXe^+^ ions are also observed in a xenon matrix (953.7, 842.3, and 730.3 cm^−1^)^34^. No KrHKr^+^ and ArHAr^+^ are observed after 250-nm photolysis of PhAc in krypton and argon matrices^35, 36^. Photolysis of PhAc-d_6_ leads to the formation of C_6_D_5_CC radicals, whose spectrum also agrees with the calculations (Fig. 3 and Table 1). Particularly characteristic is the appearance of a band at 1335 cm^−1^ that is absent after photolysis of PhAc. The broad absorption in the 1700 to 1300 cm^−1^ region is virtually unchanged upon deuteration, which confirms its electronic origin. XeDXe^+^ ions has bands at 516.2 and 634.0 cm^−1^ ^34^; however, they are close to the strong PhAc-d_6_ bands, which complicates the identification. Experimental information on the phenylethynyl radical is very limited. Gu *et al*. studied the reaction of dicarbon molecules with benzene under single collision conditions and discussed the possible involvement of phenylethynyl radical as a reaction product^37^. Kasai and McBay performed photolysis of phenyliodoacetylene in an argon matrix. The photolysis product was assigned to phenylethynyl radical based on ESR spectroscopy^38^. To the best of our knowledge, the present work provides the first infrared spectrum of phenylethynyl radical.

**Table 1:** Experimental and calculated vibrational transitions (in cm^−1^) of C_6_H_5_CC and C_6_D_5_CC radicals^a^.

Experiment			Calculated	
Xe	Kr^b^	Ar^b^	M06-2X	Assignment
n.o.	n.o.	n.o.	1505.3 (6)	CC ring stretch + CH in-plane bend
*1338.7/1335.4/1332.0*	—	—	*1377.1 (32*)	*CC ring stretch + CD in-plane bend*
n.o.	n.o.	n.o.	1220.6 (31)	C–C≡ stretch + CH in-plane bend
*863.3*	—	—	*878.8 (2)*	*C–C≡ stretch + CD in-plane bend*
1154.6	1156.4	1157.2	1172.9 (101)	C–C≡ stretch + CH in-plane bend
*1136.9/1133.4*	—	—	*1156.1 (77)*	*C–C≡ stretch + CD in-plane bend*
n.o.	n.o.	n.o.	1051.3 (0)	CH in-plane bend + CC stretch
*833.0/828.5*	—	—	*850.2 (12*)	*CD in-plane bend + CC stretch*
992.6/990.4	994.6/991.7	995.4/992.4	1015.1 (5)	CCC ring bend
*952.0*	—	—	*972.5 (2)*	*CCC ring bend*
929.2/926.6	926.8	926.8	977.3 (1)	CH out-of-plane
*771.3*	—	—	*808.0 (0)*	*CD out-of-plane*
751.9 (t)	751.3 (t)	753.9 (t)	790.0 (28)	CH out-of-plane
*642.7*	—	—	*660.8 (1)*	*CD out-of-plane*
n.o.	n.o.	n.o.	783.6 (0)	CCC ring bend + CH in-plane bend
*718.7*	—	—	*732.4 (1)*	*CCC ring bend + CD in-plane bend*
670.7/669.3/665.3	671.4/667.3	672.2/669.2	681.7 (41)	CH out-of-plane
*524.0/520.3/516.0*	—	—	*533.6 (25)*	*CD out-of-plane*

^a^C_6_D_5_CC values are in *italics*. Calculated frequencies are unscaled and infrared intensities (in km mol^−1^) are in parenthesis. (t) – tentative assignment; n.o. – not observed. ^b^No experiments with deuterated PhAc were performed in argon and krypton matrices.

Irradiation at 193 nm also decomposes PhAc in a matrix; however, the bands assigned above to phenylethynyl radical are hardly visible (lowest spectrum in Fig. 3). Instead, numerous relatively weak bands appear, suggesting the existence of other photochemical channels. Sorkhabi *et al*. investigated 193-nm photolysis of PhAc under collision-free conditions^39^. They reported the appearance of acetylene (C_2_H_2_) and C_6_H_4_ isomers (*E*/*Z*-hexene-1,5-diyne) as the primary products. Some C_6_H_4_ molecules further decomposed, via H_2_ elimination, to 1,3,5-hexatriene. Hofmann *et al*. studied pyrolysis of PhAc and identified several transient products (e.g., phenyl, phenylvinyl, and ethynylphenyl radicals)^40^. After comparison with the most intense absorptions observed after 193-nm photolysis of PhAc (583, 619, 638, 767, 1335, 1370, and 3331 cm^−1^ in Ar; 582, 617, 636, 766, 1334, 1368, and 3322 cm^−1^ in Kr; 580, 616, 634, 765, 1332, 1367, and 3314 cm^−1^ in Xe), the presence of these species, in particular, C_6_H_4_ isomers and ethynylphenyl radical, cannot be ruled out. The amount of (XeHXe)^+^ is very small compared to photolysis at 250 nm.

**Figure 4:**
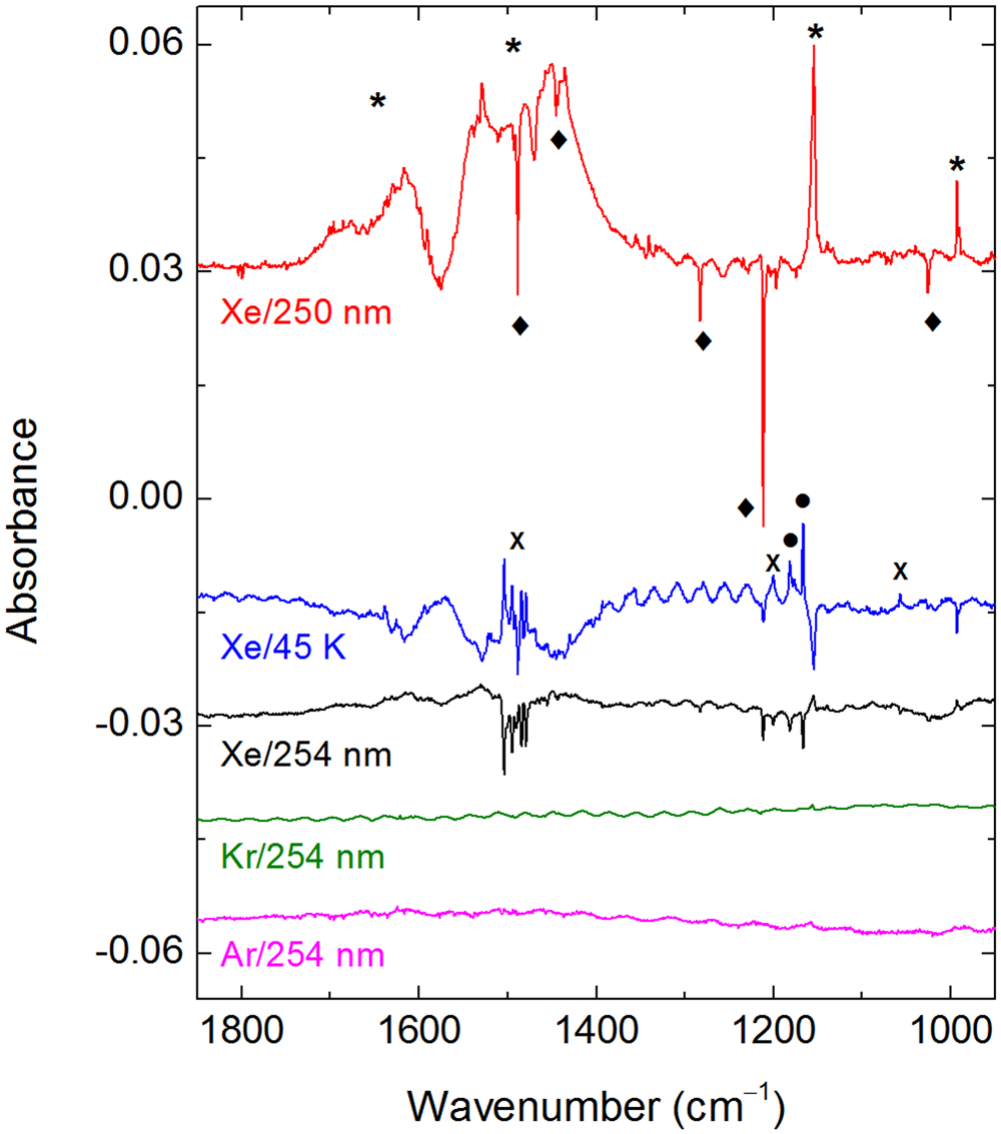
Difference FTIR spectra showing (from top to bottom) the results of 250-nm photolysis of a PhAc/Xe matrix (upper trace), of annealing at 45 K of the photolyzed sample (second upper trace), and of 254-nm irradiation (middle trace). The results of 254-nm irradiation of photolyzed and annealed (at 30 and 20 K) PhAc/Kr and PhAc/Ar matrices are also presented. The bands of PhAc (diamonds), C_6_H_5_CC radical (asterisks), C_6_H_5_CCXeH (crosses), and HXeH (circles) are marked.

Annealing of the photolyzed matrices at certain temperatures activates the mobility of H atoms^41–43^, which can promote in principle their reactions with neutral Ng–Y centers and produce HNgY molecules^11^. For a PhAc/Xe matrix photolyzed at 250 nm, annealing at about 40 K particularly leads to the formation of the known species HXeH (1181.2 and 1166.4 cm^−1^)^44^. In addition, several previously unreported bands also appear upon annealing at temperatures activating H-atom mobility (Fig. 4 and Table 2). The bands of phenylethynyl radical decrease upon the annealing indicating its reaction. No analogous bands with a normal matrix shifts are seen after photolysis and annealing of PhAc/Kr and PhAc/Ar matrices, which suggests participation of a xenon atom in the new absorber.

**Table 2:** Experimental and calculated vibrational transitions (in cm^−1^) of C_6_H_5_CCXeD and C_6_D_5_CCXeD in a xenon matrix^a^.

Experiment	Calculated	Assignment
1503.7/1500.6(sh)/1494.8/1490.2/1484.1/1479.0	1748.1 (1586)	H–Xe stretch
*1088.6/1083.3/1078.2/1072.0*	*1242.1 (732)*	*D*–*Xe stretch*
1468.5/1463.8/1460.0/1454.9 (t)	1670.2 (75)	CC ring stretch + H–Xe stretch
*n.o*.	*1637.7 (26)*	*CC* *ring stretch*
1200.1	1237.5 (93)	C–C≡ stretch
*1064.0/1061.6/1054.9/1047.0 (t)*	*1171.4 (190)*	*C*–*C≡ stretch* + *D*–*Xe stretch*
1056.9 (t)	1106.5 (5)	CH in-plane bend + CC ring stretch
*n.o*.	*831.1 (2)*	*CD in-plane bend + CC ring stretch*
908.7 (t)	951.6 (3)	CH out-of-plane bend
*762.2*	*799.2 (3)*	*CD out-of-plane bend*
776.3/773.6	796.0 (94)	CCC ring bend + C–C≡ stretch
*726.7*	*744.6 (89)*	*CCC ring bend + C–C≡ stretch*
636.3 (t)	661.1 (3)	C–Xe–H in-plane bend
*n.o*.	*481.0 (4)*	*C–Xe–D in-plane bend*
631.7 (t)	658.0 (4)	C–Xe–H out-of-plane bend
*n.o*.	*473.5 (4)*	*C–Xe–D out-of-plane bend*
510.4	525.9 (83)	CCC ring bend + CC–Xe stretch
*500.3*	*514.9 (82)*	*CCC ring bend + CC–Xe stretch*

^a^Observed after annealing at 45 K. C_6_D_5_CCXeD values are in *italic*. Calculated wavenumbers (M06-2X) are unscaled and infrared intensities (in km mol^−1^) are in parenthesis. (t) – tentative assignment; n.o. – not observed.

The group of bands at ca. 1500 cm^−1^ (the strongest component at 1503.7 cm^−1^) is assigned to the H–Xe stretching mode of C_6_H_5_CCXeH. The calculated frequencies are 1748.1 (M06-2X), 1701.4 (MP2), and 1667.7 (B3LYP) cm^−1^ (Table S4), i.e. higher than the experimental ones; however, this overestimate is typical for the harmonic calculations of noble-gas hydrides^11^. The experimental bands at ~775 and 510 cm^−1^ are assigned to the CCC ring bending modes, in agreement with the calculated values of 796.0 and 525.9 cm^−1^ (M06-2X). Some other (less certain) assignments are shown in Table 2. Consistently, no bands assigned to C_6_H_5_CCXeH appear in the experiments with 193-nm photolysis, producing negligible amount of phenylethynyl radicals.

The noble-gas hydrides are very photolabile species and their identification can be supported by the selective photodecomposition^14, 15, 17, 45^. Accordingly, 254-nm irradiation by a mercury lamp mainly decomposes the bands of HXeH and the bands assigned above to C_6_H_5_CCXeH. The bands assigned to C_6_H_5_CCXeH are bleached with similar rates, suggesting that they belong to the same species. The bands assigned to phenylethynyl radical are partially recovered by 254-nm irradiation, mainly due to decomposition of C_6_H_5_CCXeH. In fact, production of phenylethynyl radical from PhAc by the mercury lamp is relatively inefficient. Indeed, no formation of phenylethynyl radicals is observed at this irradiation of a PhAc/Xe matrix (without 250-nm photolysis). Moreover, in argon and krypton matrices, only minor amounts of phenylethynyl radicals are produced by the mercury lamp after 250-nm photolysis and annealing (Fig. 4). It should be noted that the small decrease of the PhAc bands seen in Fig. 4 (middle trace) is mainly an artefact due to inaccurate positioning of the matrix with respect to the infrared beam of the spectrometer.

It should be noted that the experimental H–Xe stretching frequency of C_6_H_5_CCXeH is very close to that of HXeCCH. However, the formation of significant amounts of HXeCCH in these experiments is ruled out based on non-observation of the CH stretching (3273 cm^−1^) and CCH bending (626 cm^−1^) bands of this molecule. To recall, the calculations predict very similar H–Xe stretching frequencies of HXeCCH and C_6_H_5_CCXeH but the other fundamentals are essentially different.

**Figure 5:**
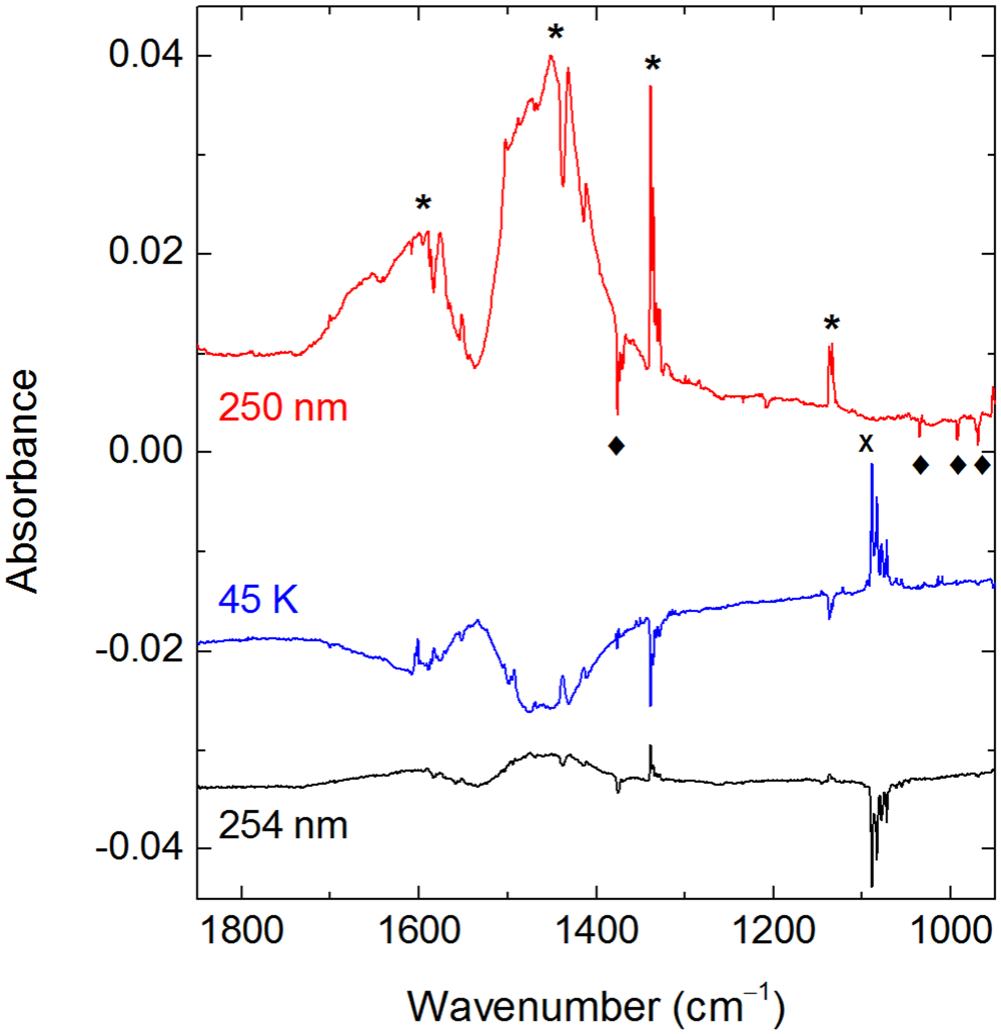
Difference FTIR spectra showing the results of 250-nm photolysis of a PhAc-d_6_/Xe matrix (upper trace), of annealing of the previous matrix at 45 K (middle trace), and of 254-nm irradiation of the previous matrix (lowest trace). The bands of PhAc-d_6_ (diamonds), C_6_D_5_CC radical (asterisks), and C_6_D_5_CCXeD (crosses) are marked.

The experiments with PhAc-d_6_ fully support the assignment of C_6_H_5_CCXeH. The D–Xe stretching and two ring bending modes are at ~1080 (multiple bands with the strongest component at 1088.6 cm^−1^), 727, and 500 cm^−1^, giving H/D frequency ratios of 1.381, 1.07, and 1.02, respectively (Fig. 5 and Table 2). The calculations lead to the comparable H/D frequency ratios of 1.41, 1.07, and 1.02 (M06-2X). The experimental H/D frequency ratio for the H–Xe stretching mode of HXeCCH (1.379) is very similar to the present one^14^.

We also performed experiments with PhAc-d_1_/Xe matrices (partially deuterated PhAc C_6_H_5_CCD) and obtained remarkable results. The D–Xe stretching mode presumably of C_6_H_5_CCXeD appear at frequencies 1089.6, 1084.1, 1079.3, and 1073.0 cm^−1^ that are very similar to those obtained in a PhAc-d_6_/Xe matrix. As a less expected result, in addition to the bands of DXeD, the bands of HXeH and HXeD are observed as well as a small amount of C_6_H_5_CCXeH (Fig. S2 in the Supplementary Information). It follows that 250-nm photolysis of PhAc-d_1_ produces some amounts of H atoms by their detachment from the ring or by a more complex mechanism.

This observation makes us to consider the photoproduction of other species, in addition to phenylethynyl radical. Indeed, 250-nm light can detach H atoms from the ring of PhAc to produce C_6_H_4_CCH^46^. However, the bands assigned to phenylethynyl radical (Table 1 and Fig. 3) cannot originate from C_6_H_*n*_CCH (*n* < 5) species, because the latter are not expected to have low-energy electronic transitions and to lead to a noble-gas hydride upon annealing. More interesting is to consider the formation of C_6_H_4_CC (triplet) species with two detached H atoms. In this situation, C_6_H_4_CCXeH is a possible species, with the H–Xe stretching frequency, similar to that of C_6_H_5_CCXeH (Tables S4 and S7 and Fig. S1 in the Supplementary Information). However, we have only one set of experimental bands produced by photolysis, decreasing upon annealing, and partially recovering in a xenon matrix by 254-nm irradiation. For example, the strongest vibrational band at 1155 cm^−1^ (C–C≡ stretch + CH in-plane bend) shows no splitting. On the other hand, the calculations suggest that this mode absorbs at different frequencies for C_6_H_5_CC and C_6_H_4_CC (Tables S6 and S7 in the Supplementary Information). The other transitions of C_6_H_4_CC are predicted to depend on the position of the detached H atom (*o−*, *m−*, and *p−*; Table S7 and Fig. S1 in the Supplementary Information). Furthermore, the calculated frequencies of C_6_H_5_CCXeH and C_6_H_4_CCXeH are also quite distinguishable (except the H–Xe stretch; Table S7 in the Supplementary Information). Thus, the only alternative to our assignment is the exclusive formation of C_6_H_4_CC and C_6_H_4_CCXeH with the same position of the detached H atom and the absence of C_6_H_5_CC and C_6_H_5_CCXeH. We consider this possibility as improbable and suggest that 250-nm photolysis of matrix-isolated PhAc mainly leads to phenylethynyl radical and some C_6_H_4_CCH species and C_6_H_5_CCXeH is the main product of annealing in a xenon matrix.

**Figure 6:**
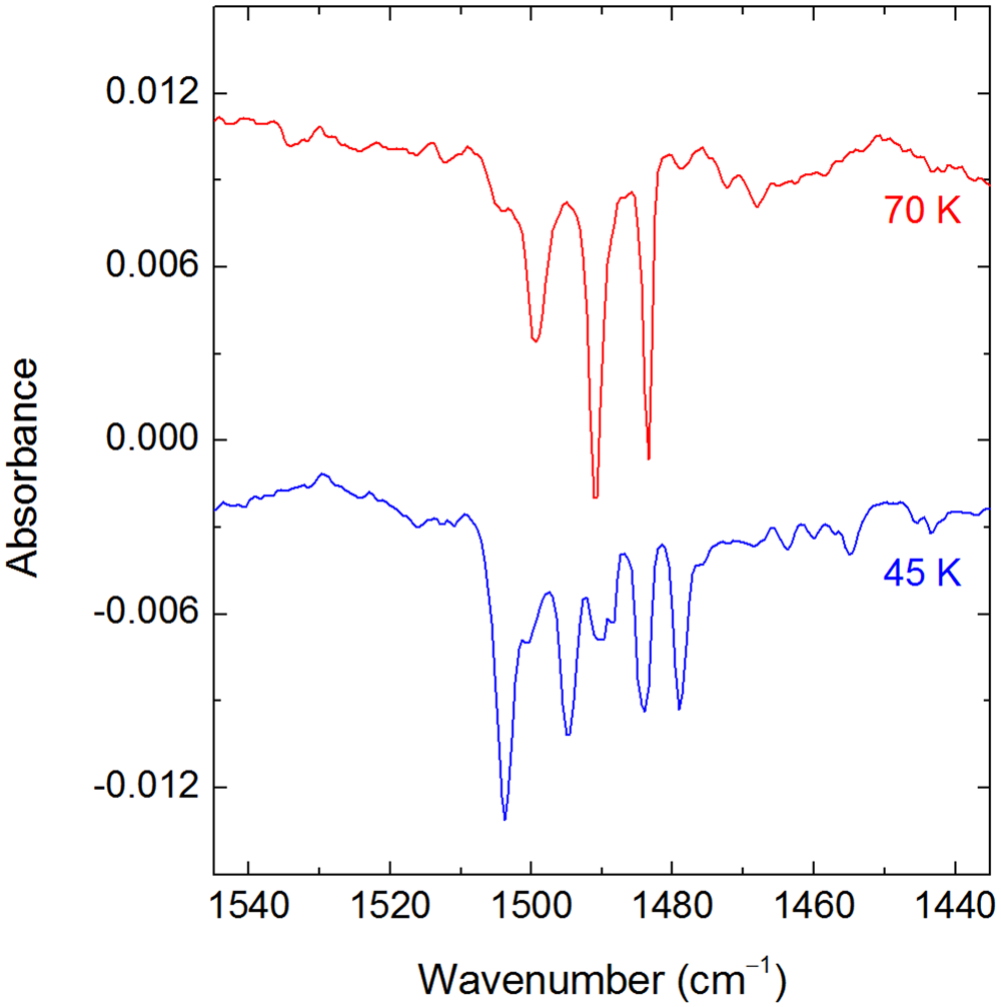
Difference FTIR spectra showing the result of 254-nm irradiation of PhAc/Xe matrices photolyzed at 250 nm and then annealed at 70 K (upper trace) or at 45 K (lower trace). The negative bands are assigned to the H–Xe stretching mode of C_6_H_5_CCXeH.

In the next experiments, the PhAc/Xe matrices were photolyzed at 250 nm and then annealed at temperatures up to 70 K. After annealing at 70 K, the bands assigned to C_6_H_5_CCXeH were still observed, which indicates its stability at this temperature. The high-temperature annealing changes the structure of the H–Xe stretching absorption and the component at 1490 cm^−1^ becomes the strongest one (Fig. 6). A similar effect was reported for HXeC_4_H^17^, and it suggests the matrix-site nature of the observed splitting of the H–Xe stretching bands. It should be noted that the matrix-site splitting is often observed for the H–Xe stretching band of noble-gas hydrides, which is due to the sensitivity of this vibration to the local matrix morphology. The mechanisms of this splitting was studied in detail for HArF in an argon matrix^47^.

## Conclusions

The aromatic noble-gas hydride, C_6_H_5_CCXeH, was prepared by 250-nm photolysis of phenylacetylene (C_6_H_5_CCH, PhAc) isolated in a xenon matrix and subsequent thermal mobilization of H atoms at about 40 K. C_6_H_5_CCXeH is formed in the C_6_H_5_CC + Xe + H reaction of the neutral fragments. This reaction is possible at low temperatures because C_6_H_5_CCXeH is computationally lower in energy than the C_6_H_5_CC + Xe + H fragments by 0.60 eV at the M06-2X/aug-cc-pVTZ-PP level of theory. No similar compounds with krypton and argon were obtained, which is in agreement with the calculated energetics.

The characteristic H–Xe stretching mode of C_6_H_5_CCXeH is observed at ~1500 cm^−1^. This assignment is confirmed by experiments with fully deuterated phenylacetylene (PhAc-d_6_) leading to C_6_D_5_CCXeD with the D–Xe stretching absorption at ~1080 cm^−1^, giving an H/D frequency ratio of 1.381. A number of other fundamentals of C_6_H_5_CCXeH and C_6_D_5_CCXeD were also identified showing characteristic shifts upon deuteration. The experimental assignment is fully supported by extensive quantum chemical calculations at different levels of theory.

The preparation of C_6_H_5_CCXeH demonstrates the possibility of new synthetic approaches in noble-gas chemistry, which is currently dominated by compounds with noble-gas atoms bound to halogens. C_6_H_5_CCXeH is the first aromatic noble-gas hydride and to our knowledge, the first halogen-free aromatic Ng compound, thus, opening new perspectives in this field.

## Materials and Methods

### Computational details

The quantum chemical calculations were performed at the DFT (with the B3LYP^48^, CAM-B3LYP^49^, M06-2X^50, 51^, and wB97XD^52^ functionals) and MP2^53^ levels of theory. H, C, Ar and Kr atoms were described by the standard aug-cc-pVTZ basis set^54^. For Xe atoms, the basis set combined with an effective core pseudopotential (aug-cc-pVTZ-PP) was used^55^. The pseudopotential was taken from the EMSL Basis Set Library^56, 57^. The calculations were carried out using the Gaussian 09 (revision E.01) program^58^. The geometry optimizations were followed by harmonic frequency calculations at the same level of theory, which also gave the zero-point vibrational energies (ZPVE) and verified the nature of the obtained minima. The DFT calculations employed an ultrafine integration grid and very tight optimization convergence criteria, and the MP2 calculations used tight optimization convergence criteria. The atomic charges were obtained using the natural population analysis (NPA)^59^ as implemented in the Gaussian program. CCSD(T)^60–62^ single-point energy evaluations were carried out on the MP2 optimized geometries (Table S3 in the Supplementary Information).

### Experimental details

The PhAc/Ng (Ng = Ar, Kr, and Xe), PhAc-d_1_/Xe, and PhAc-d_6_/Xe mixtures were prepared with typical concentration ratios of 1/1000. PhAc (≥98%, Sigma-Aldrich), PhAc-d_1_ (≥99%, deuteration 99%, Sigma-Aldrich) and PhAc-d_6_ (≥99%, deuteration 95%, Sigma-Aldrich) were degassed by several freeze-pump-thaw cycles. Argon (≥99.9999%, AGA), krypton (≥99.999%, Linde) and xenon (≥99.999%, Linde) were used without further purification. The gas mixtures were deposited onto a CsI window held at 15, 20, and 35 K for argon, krypton, and xenon matrices, respectively, in a closed-cycle helium cryostat (DE-202A, APD). The matrix thickness was ~100 *μ*m. The FTIR spectra in the 4000–500 cm^−1^ range were measured at 9 K with a Nicolet 60 SX spectrometer by co-adding 500 scans at a spectral resolution of 1 cm^−1^. Photolysis of the matrix-isolated species was performed at 9 K using an optical parametric oscillator (Continuum, OPO Sunlite) at 250 nm with a pulse energy of ~5 mJ and a repetition rate of 10 Hz. 193-nm photolysis by an excimer laser (MSX-250, MPB, ~10 mJ cm^−2^, 1 Hz) was also tested. After photolysis, the matrices were annealed (for ~5 min) at different temperatures and then cooled down to 9 K for spectral measurements. The annealed matrices were irradiated with a low-pressure mercury lamp (254 nm, HG-1, Ocean Optics).

### Data availability

All essential data generated or analyzed during this study are included in this published article (and its Supplementary Information). Additional information is available from the corresponding author on reasonable request.

## Electronic supplementary material

Supplementary Information
