# Supplementary material for: An aromatic noble-gas hydride: C6H5CCXeH
Source: Sci Rep. 2017 Jun 9;7:3130. doi: 10.1038/s41598-017-02869-9 (PMC5466666; doi:10.1038/s41598-017-02869-9)
Supplement: Supplementary file 1 — Supplementary Information [file 41598_2017_2869_MOESM1_ESM.pdf]

Supplementary Information for:

An aromatic noble-gas hydride: C_6H_5CCXeH.

*Luís Duarte and Leonid Khriachtchev**

Department of Chemistry, University of Helsinki, P.O. Box 55, FI-00014 Helsinki, Finland

***leonid.khriachtchev@helsinki.fi**

Contents

Figure S1. Structure of possible products of photolysis and annealing. 3

Figure S2. Difference FTIR spectra showing the results of 250-nm photolysis of a PhAc-d_1/Xe matrix, of annealing of the photolyzed matrix at 45 K, and of 254-nm irradiation of the previous matrix. 4

Figure S3. Atom numbering scheme for C_6H_5CCXeH and C_6H_5CCH. 5

Table S1. Calculated bond lengths of C_6H_5CCXeH and C_6H_5CCH. 6

Table S2. Calculated atomic charges in C_6H_5CCXeH and C_6H_5CCH. 8

Table S3. Calculated relative energies of noble-gas hydrides resulting from the insertion of a Ng atom (Ar, Kr and Xe) into PhAc. 10

Table S4. Calculated vibrational transitions and infrared intensities of C_6H_5CCXeH, C_6D_5CCXeD, and C_6H_5CCXeD. 11

Table S5. Calculated vibrational transitions and infrared intensities of PhAc (C_6H_5CCH), PhAc-d_6 (C_6D_5CCD), and PhAc-d_1 (C_6H_5CCD). 16

Table S6. Calculated vibrational transitions and infrared intensities of C_6H_5CC and C_6D_5CC radicals. 21

Table S7. Calculated vibrational transitions and infrared intensities of possible photolysis and annealing products at the M06-2X/aug-cc-pVTZ level. 24

Table S8. Calculated NICS at the M06-2X/aug-cc-pVTZ level. 38

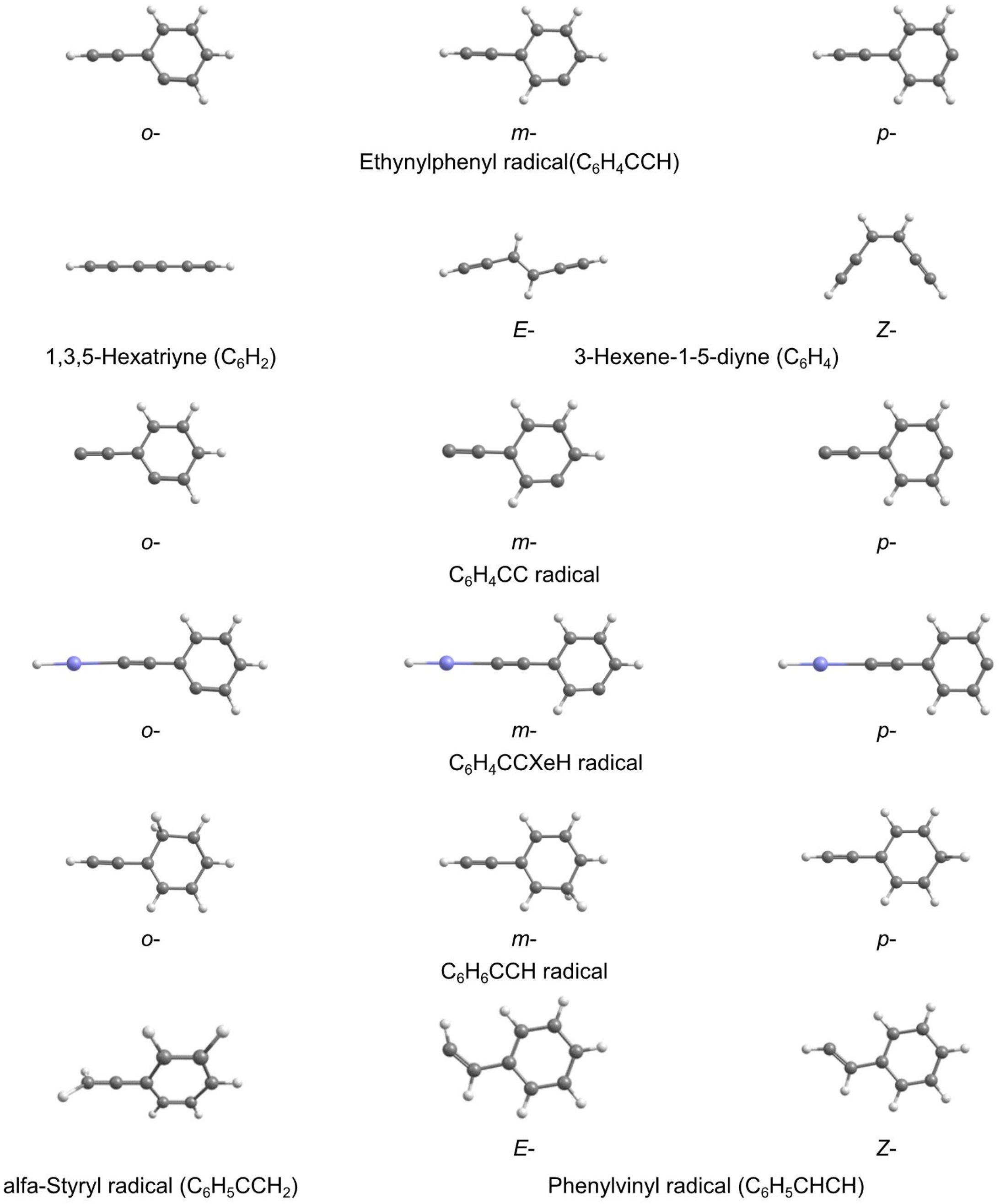

Figure S1. Structure of possible products of photolysis and annealing.

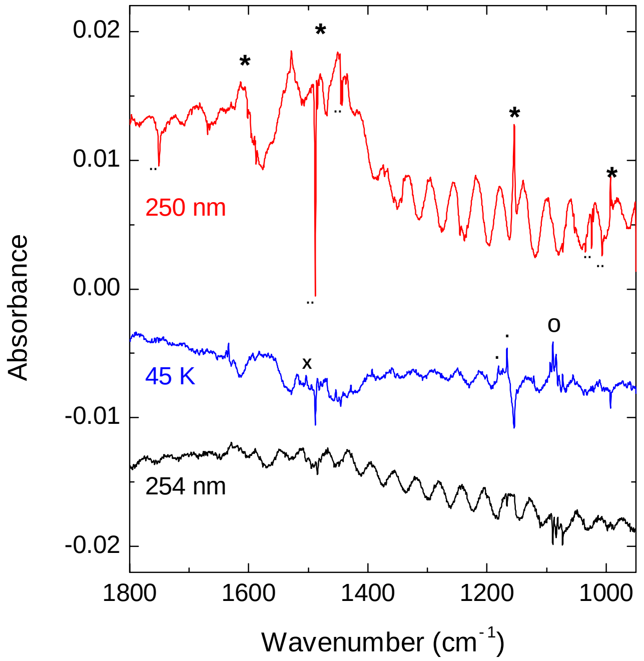

Figure S2. Difference FTIR spectra showing (from top to bottom) the results of 250-nm photolysis (70 min) of a PhAc-d_1/Xe matrix (red trace), of annealing of the photolyzed matrix at 45 K (blue trace), and of 254-nm irradiation of the previous matrix (black trace). The bands are marked for PhAc-d_1 (diamonds), C_6H_5CC radical (asterisks), C_6H_5CCXeH (small amount; crosses), C_6H_5CCXeD (and HXeD at 1093 cm^{-1}; open circles) and HXeH (solid circles).

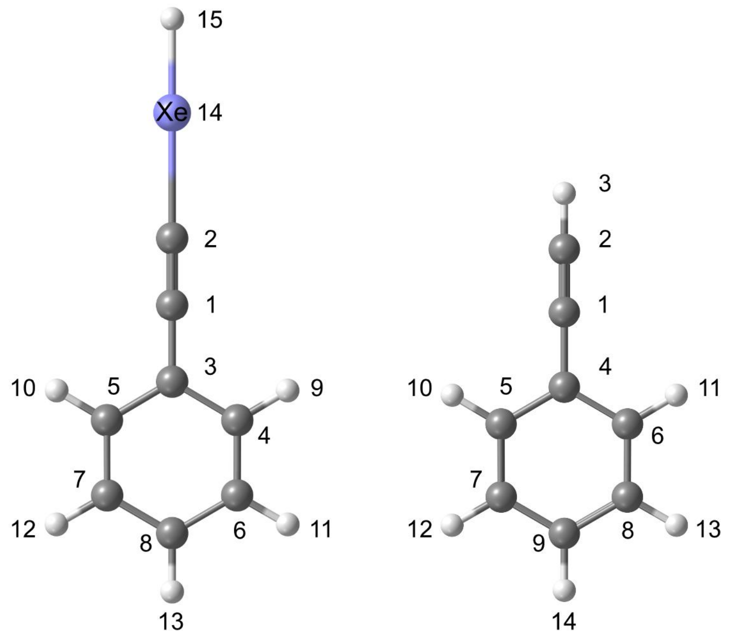

Figure S3. Atom numbering scheme for C_6H_5CCXeH and C_6H_5CCH.

Table S1. Calculated bond lengths (R, in angstroms) of C_6H_5CCXeH and C_6H_5CCH.[a]

			C_6H_5CCXeH		
**Bond**	**M06-2X**	**B3LYP**	**CAM-B3LYP**	**wB97XD**	**MP2**
R(1-2)	1.214	1.217	1.210	1.213	1.237
R(1-3)	1.433	1.427	1.430	1.431	1.425
R(2-14)	2.348	2.359	2.347	2.336	2.326
R(3-4)	1.397	1.403	1.395	1.397	1.404
R(3-5)	1.397	1.403	1.395	1.397	1.404
R(4-6)	1.385	1.387	1.382	1.384	1.391
R(4-9)	1.081	1.081	1.080	1.081	1.082
R(5-7)	1.385	1.387	1.382	1.384	1.391
R(5-10)	1.081	1.081	1.080	1.081	1.082
R(6-8)	1.389	1.391	1.385	1.387	1.395
R(6-11)	1.082	1.082	1.081	1.082	1.082
R(7-8)	1.389	1.391	1.385	1.387	1.395
R(7-12)	1.082	1.082	1.081	1.082	1.082
R(8-13)	1.081	1.082	1.081	1.081	1.082
R(14-15)	1.738	1.765	1.740	1.738	1.739

C_6H_5CCH					
**Bond**	**M06-2X**	**B3LYP**	**CAM-B3LYP**	**wB97XD**	**MP2**
R(1-2)	1.199	1.202	1.195	1.198	1.219
R(1-4)	1.432	1.426	1.429	1.430	1.427
R(2-3)	1.063	1.061	1.062	1.062	1.062
R(4-5)	1.396	1.402	1.394	1.395	1.402
R(4-6)	1.396	1.402	1.394	1.395	1.402
R(5-7)	1.385	1.387	1.382	1.384	1.391
R(5-10)	1.081	1.081	1.080	1.081	1.082
R(6-8)	1.385	1.387	1.382	1.384	1.391
R(6-11)	1.081	1.081	1.080	1.081	1.082
R(7-9)	1.388	1.391	1.385	1.387	1.394
R(7-12)	1.081	1.082	1.081	1.081	1.082
R(8-9)	1.388	1.391	1.385	1.387	1.394
R(8-13)	1.081	1.082	1.081	1.081	1.082
R(9-14)	1.081	1.081	1.081	1.081	1.082

[a] Atom numbering is given in Figure S3. The calculations were performed with the aug-cc-pVTZ-PP basis set.

Table S2. Calculated atomic charges (in elementary charges) in C_6H_5CCXeH and C_6H_5CCH.[a]

		C_6H_5CCXeH			
**Atom**	**M06-2X**	**B3LYP**	**CAM-B3LYP**	**wB97XD**	**MP2**
C_1	-0.159	-0.148	-0.151	-0.150	-0.150
C_2	-0.406	-0.397	-0.420	-0.425	-0.504
C_3	-0.124	-0.120	-0.118	-0.119	-0.122
C_4	-0.170	-0.167	-0.170	-0.170	-0.154
C_5	-0.170	-0.167	-0.170	-0.170	-0.154
C_6	-0.207	-0.202	-0.205	-0.205	-0.193
C_7	-0.207	-0.202	-0.205	-0.205	-0.193
C_8	-0.209	-0.205	-0.208	-0.207	-0.195
H_9	+0.213	+0.208	+0.212	+0.212	+0.201
H_{10}	+0.213	+0.208	+0.212	+0.212	+0.201
H_{11}	+0.208	+0.203	+0.206	+0.207	+0.194
H_{12}	+0.208	+0.203	+0.206	+0.207	+0.194
H_{13}	+0.207	+0.202	+0.205	+0.206	+0.194
Xe_{14}	+0.705	+0.701	+0.724	+0.729	+0.806
H_{15}	-0.105	-0.121	-0.119	-0.121	-0.126

C_6H_5CCH					
**Atom**	**M06-2X**	**B3LYP**	**CAM-B3LYP**	**wB97XD**	**MP2**
C_1	-0.023	-0.019	-0.018	-0.019	-0.019
C_2	-0.206	-0.207	-0.211	-0.212	-0.217
H_3	+0.232	+0.229	+0.231	+0.233	+0.230
C_4	-0.138	-0.135	-0.135	-0.136	-0.145
C_5	-0.163	-0.159	-0.162	-0.162	-0.139
C_6	-0.163	-0.159	-0.162	-0.162	-0.139
C_7	-0.204	-0.200	-0.203	-0.204	-0.192
C_8	-0.204	-0.200	-0.203	-0.204	-0.192
C_9	-0.198	-0.194	-0.196	-0.196	-0.176
H_{10}	+0.217	+0.212	+0.215	+0.215	+0.202
H_{11}	+0.217	+0.212	+0.215	+0.215	+0.202
H_{12}	+0.212	+0.207	+0.210	+0.210	+0.195
H_{13}	+0.212	+0.207	+0.210	+0.210	+0.195
H_{14}	+0.210	+0.205	+0.209	+0.209	+0.194

[a] Atom numbering is given in Figure S3. The calculations were performed with the aug-cc-pVTZ-PP basis set.

Table S3. Calculated relative energies (in eV) of noble-gas hydrides resulting from the insertion of a Ng atom (Ar, Kr and Xe) into PhAc. [a]

**Level of Theory**	**E(HNgY)-E(H+Ng+Y)**			**E(HNgY)-E(HY+Ng)**		
	**Ar**	**Kr**	**Xe**	**Ar**	**Kr**	**Xe**
**CCSD(T)**	0.2	- 0.6	- 1.6	6.4	5.6	4.7
**MP2**[b]	- 0.5	- 1.4	- 2.4	6.4	5.5	4.5
**B3LYP**	0.9	0.2	- 0.7	6.3	5.5	4.7
**CAM-B3LYP**	1.1	0.3	- 0.6	6.6	5.8	4.9
**M06-2X**	1.0	0.2	- 0.6	6.6	5.8	4.9
**wB97XD**	2.4	0.3	- 0.7	7.8	5.8	4.8

[a] The CCSD(T) energy values were obtained after single-point energy evaluations on the MP2 geometries (without ZPVE correction). All other energy values include ZPVE correction.
[b] Single-reference MP2 calculations can produce an inaccurate energy diagram for the formation of noble-gas hydrides. See Lignell, A., Khriachtchev, L., Lundell, J., Tanskanen, H. & Räsänen, M. On theoretical predictions of noble-gas hydrides. *J. Chem. Phys.* **125**, 184514 (2006).

Table S4. Calculated vibrational transitions (ω, in cm^{-1}) and infrared intensities (I, in km mol^{-1}) of C_6H_5CCXeH, C_6D_5CCXeD, and C_6H_5CCXeD.

C_6H_5CCXeH									
**M06-2X**		**B3LYP**		**CAM-B3LYP**		**wB97XD**		**MP2**	
ω	I	ω	I	ω	I	ω	I	ω	I
3227.8	8	3193.8	8	3217.0	6	3221.0	9	3227.9	13
3222.6	13	3189.4	20	3212.4	15	3215.6	21	3220.1	16
3216.6	10	3182.4	18	3205.3	15	3208.2	17	3214.1	5
3206.0	3	3169.9	6	3193.5	5	3197.4	4	3203.4	1
3197.8	1	3161.7	2	3185.0	2	3188.4	1	3196.3	1
2245.3	12	2194.5	12	2251.6	15	2242.5	17	2083.2	10
1748.1	1586	1667.7	1439	1765.1	1616	1787.3	1612	1701.4	1796
1670.2	75	1635.9	169	1677.4	70	1674.4	57	1632.2	60
1640.4	1	1606.3	2	1646.8	2	1644.1	2	1606.2	2
1533.1	57	1523.3	53	1546.1	58	1539.9	55	1508.1	49
1484.6	4	1475.2	3	1495.9	4	1489.6	4	1476.7	2
1347.4	0	1355.1	0	1364.3	0	1359.1	0	1439.4	1
1301.8	0	1307.5	0	1308.8	0	1314.2	0	1324.0	0
1237.5	93	1227.1	87	1243.6	91	1239.3	91	1227.5	95
1197.0	1	1199.2	3	1207.8	1	1208.5	1	1189.1	1
1173.2	0	1180.6	0	1183.3	0	1185.9	0	1170.6	0
1106.5	5	1099.4	5	1112.4	6	1110.8	6	1091.1	6
1060.9	11	1049.9	10	1065.6	11	1062.4	12	1046.0	14
1025.0	0	1018.0	1	1036.4	0	1028.9	0	1012.7	0
1020.5	0	1013.4	0	1032.9	0	1027.5	0	984.6	0
1005.6	0	995.5	0	1017.5	0	1009.9	0	971.0	0
951.6	3	939.8	3	961.4	4	955.1	3	919.3	3
871.9	0	861.7	0	878.9	0	875.5	0	852.3	0
796.0	94	791.6	78	801.4	95	798.7	97	789.1	80
786.5	44	780.2	41	795.5	40	790.7	44	761.4	55
711.8	32	711.0	34	722.9	36	718.7	35	688.1	20

C_6H_5CCXeH									
**M06-2X**		**B3LYP**		**CAM-B3LYP**		**wB97XD**		**MP2**	
ω	*I*	*ω*	*I*	*ω*	*I*	*ω*	*I*	*ω*	*I*
661.1	3	643.5	4	670.4	3	677.1	4	670.6	4
658.0	4	639.7	5	667.9	5	674.9	5	669.7	5
634.9	0	637.2	0	644.5	0	642.2	0	622.4	0
551.2	8	547.8	8	560.1	8	554.9	8	531.2	8
537.8	1	528.8	1	542.7	1	538.6	1	521.5	68
525.9	83	525.1	67	531.0	88	529.4	90	505.2	1
410.7	0	413.9	0	420.4	0	416.0	0	397.3	0
364.4	0	359.7	0	372.9	0	367.7	0	348.5	0
170.0	3	170.1	2	181.7	3	177.9	3	167.4	56
163.3	65	159.7	55	161.5	66	161.1	67	160.4	2
150.9	1	148.6	1	159.2	2	157.6	2	148.1	1
36.0	5	40.2	4	43.5	6	44.3	6	40.3	5
31.5	6	37.9	5	43.2	4	41.8	4	40.0	4

C_6D_5CCXeD									
**M06-2X**		**B3LYP**		**CAM-B3LYP**		**wB97XD**		**MP2**	
ω	*I*	*ω*	*I*	*ω*	*I*	*ω*	*I*	*ω*	*I*
2394.1	5	2367.2	6	2385.8	5	2389.1	7	2394.8	7
2386.6	8	2360.1	12	2378.7	10	2381.5	12	2384.9	9
2379.2	3	2352.7	6	2371.2	5	2373.3	5	2376.8	2
2367.2	1	2339.7	2	2358.7	2	2361.3	2	2364.1	1
2357.4	1	2330.8	2	2348.9	2	2350.8	1	2355.8	1
2245.2	10	2194.3	9	2251.5	13	2242.4	15	2083.0	8
1637.7	26	1600.5	27	1643.0	27	1640.0	26	1600.1	22
1603.7	0	1565.3	0	1608.0	0	1605.7	0	1570.0	1
1424.7	39	1402.8	38	1431.8	38	1426.2	35	1456.3	0
1367.3	1	1354.5	0	1374.1	1	1368.2	1	1397.0	37
1295.9	0	1302.5	0	1301.6	0	1308.6	0	1330.6	0

C_6D_5CCXeD									
**M06-2X**		**B3LYP**		**CAM-B3LYP**		**wB97XD**		**MP2**	
ω	*I*	*ω*	*I*	*ω*	*I*	*ω*	*I*	*ω*	*I*
1242.1	732	1187.9	478	1254.1	752	1269.4	769	1210.0	763
1171.4	190	1160.2	411	1178.3	179	1173.7	155	1159.2	258
1050.9	0	1057.2	0	1065.5	0	1059.6	0	1039.6	0
977.6	0	974.8	1	988.6	0	983.5	0	969.5	0
883.5	7	884.1	4	890.9	6	890.4	6	878.7	5
856.5	2	857.0	1	875.2	1	864.1	0	850.8	1
856.4	0	854.9	1	862.3	1	862.9	1	847.9	0
854.8	1	851.0	0	861.9	1	860.2	1	822.8	3
831.1	2	835.4	3	840.1	3	840.0	3	798.9	0
819.4	0	810.7	0	829.2	0	822.5	0	789.7	0
799.2	3	789.6	3	808.2	3	802.0	3	767.5	3
744.6	89	741.4	75	749.7	91	747.9	92	738.0	78
678.6	0	670.4	0	683.9	0	681.1	0	663.0	0
660.9	5	654.5	4	666.0	5	663.1	5	645.2	4
610.4	0	612.4	0	619.4	0	617.1	0	597.3	0
570.9	16	566.2	17	575.9	16	573.6	17	559.7	19
533.3	0	520.5	0	539.1	0	535.3	0	510.0	68
514.9	82	514.5	67	520.1	87	518.6	90	504.9	1
501.0	22	488.6	25	508.2	23	508.8	20	498.2	11
481.0	4	470.6	5	486.5	4	491.0	5	482.7	4
473.5	4	467.2	0	480.1	4	480.4	7	469.0	12
356.6	0	359.5	0	364.9	0	361.3	0	345.6	0
339.6	1	336.9	1	347.8	1	343.4	1	326.7	1
161.0	2	161.6	2	172.3	3	168.9	3	164.2	53
160.2	61	156.8	52	158.5	63	158.1	64	152.7	2
142.3	1	140.5	1	150.3	2	148.9	1	140.1	1
34.1	4	38.2	4	41.8	5	42.5	5	38.7	5
30.2	6	36.4	5	41.1	4	39.8	4	38.0	4

C_6H_5CCXeD									
**M06-2X**		**B3LYP**		**CAM-B3LYP**		**wB97XD**		**MP2**	
ω	*I*	*ω*	*I*	*ω*	*I*	*ω*	*I*	*ω*	*I*
3227.8	8	3193.8	8	3217.0	6	3221.0	9	3227.9	13
3222.6	13	3189.4	20	3212.4	15	3215.6	21	3220.1	16
3216.6	10	3182.4	18	3205.3	15	3208.2	17	3214.1	5
3206.0	3	3169.9	6	3193.5	5	3197.4	4	3203.4	1
3197.8	1	3161.7	2	3185.0	2	3188.4	1	3196.3	1
2245.3	10	2194.4	9	2251.5	13	2242.5	15	2083.1	8
1670.8	25	1637.2	25	1678.0	26	1674.8	25	1632.6	20
1640.4	1	1606.3	2	1646.8	2	1644.1	2	1606.2	2
1533.3	37	1523.5	34	1546.3	38	1540.1	37	1508.2	33
1484.6	4	1475.2	3	1495.9	4	1489.6	4	1476.7	2
1347.4	0	1355.1	0	1364.3	0	1359.1	0	1439.3	1
1301.8	0	1307.5	0	1308.8	0	1314.2	0	1324.0	0
1252.8	235	1230.3	1	1262.5	331	1273.8	550	1234.2	30
1224.9	671	1199.3	0	1233.5	584	1233.8	356	1200.7	971
1197.0	3	1180.6	0	1207.8	3	1208.5	3	1189.1	6
1173.2	0	1179.3	880	1183.3	0	1185.9	0	1170.6	0
1106.5	5	1099.4	5	1112.4	6	1110.8	6	1091.1	5
1060.8	12	1049.9	12	1065.6	13	1062.4	14	1046.0	17
1025.0	0	1018.0	1	1036.4	0	1028.9	0	1012.7	0
1020.5	1	1013.4	0	1032.9	0	1027.5	0	984.6	0
1005.6	0	995.5	0	1017.5	0	1009.9	0	971.0	0
951.6	3	939.8	3	961.4	4	955.1	3	919.3	3
871.9	0	861.7	0	878.9	0	875.5	0	852.3	0
795.7	100	791.4	83	801.1	102	798.4	103	788.8	86
786.5	44	780.2	41	795.5	40	790.7	44	761.3	55
711.8	32	711.0	34	722.9	36	718.7	35	688.1	21
635.4	0	637.8	0	645.0	0	642.5	0	622.5	0

C_6H_5CCXeD									
**M06-2X**		**B3LYP**		**CAM-B3LYP**		**wB97XD**		**MP2**	
ω	*I*	*ω*	*I*	*ω*	*I*	*ω*	*I*	*ω*	*I*
553.9	10	548.7	9	562.9	11	557.5	10	532.4	10
544.8	0	532.5	0	550.4	0	546.1	0	521.4	70
525.8	84	525.1	68	530.9	89	529.3	92	511.8	0
487.5	1	473.5	3	494.5	2	499.1	2	494.5	2
483.1	4	472.0	5	488.8	4	493.7	5	488.8	4
410.7	0	413.9	0	420.4	0	416.0	0	397.3	0
360.7	0	356.7	0	368.7	0	364.0	0	346.3	0
166.1	2	166.4	2	177.3	2	173.7	2	167.3	55
163.2	64	159.6	55	161.3	65	161.0	67	156.9	2
148.6	1	146.5	1	156.6	1	155.0	1	145.9	1
35.4	5	39.5	4	42.8	6	43.6	5	39.6	5
31.0	6	37.2	5	42.5	4	41.2	4	39.4	4

Table S5. Calculated vibrational transitions (ω, in cm^{-1}) and infrared intensities (I, in km mol^{-1}) of PhAc (C_6H_5CCH), PhAc-d_6 (C_6D_5CCD), and PhAc-d_1 (C_6H_5CCD).

C_6H_5CCH									
**M06-2X**		**B3LYP**		**CAM-B3LYP**		**wB97XD**		**MP2**	
ω	I	ω	I	ω	I	ω	I	ω	I
3482.7	93	3467.1	94	3478.0	98	3478.7	90	3478.3	80
3230.4	4	3196.9	4	3220.0	4	3224.1	5	3230.7	7
3225.6	9	3192.8	15	3215.5	11	3218.9	15	3223.5	12
3218.9	7	3185.4	13	3208.0	10	3211.1	12	3216.7	5
3210.3	2	3175.0	5	3198.3	3	3202.1	3	3207.7	1
3201.8	0	3166.3	0	3189.4	0	3192.8	0	3200.1	0
2251.7	8	2204.6	12	2252.3	10	2242.7	10	2119.2	1
1673.3	2	1639.7	2	1680.3	2	1676.9	2	1637.8	4
1644.0	1	1609.7	1	1650.2	1	1647.3	1	1611.3	2
1535.1	15	1525.5	14	1547.9	15	1541.7	16	1512.6	14
1487.0	5	1477.3	4	1498.1	5	1491.7	5	1477.0	3
1349.8	0	1357.4	0	1366.7	0	1361.5	0	1443.4	1
1305.7	1	1311.7	0	1312.7	1	1318.0	1	1328.6	0
1233.2	1	1223.4	1	1239.0	1	1234.4	1	1224.8	2
1198.8	0	1201.0	0	1209.7	0	1210.3	0	1192.2	0
1175.8	0	1183.1	0	1185.7	0	1188.4	0	1173.3	0
1109.1	5	1102.1	5	1115.0	5	1113.4	6	1094.4	5
1061.0	4	1050.4	4	1065.9	4	1062.7	5	1047.1	5
1027.8	0	1019.0	0	1040.3	0	1032.6	0	1013.4	0
1021.6	0	1017.6	0	1033.9	0	1028.6	0	986.6	0
1007.0	0	997.8	0	1019.5	0	1011.8	0	973.2	0
955.8	3	945.3	4	966.3	4	959.8	4	920.3	4
873.4	0	863.6	0	880.6	0	877.0	0	854.5	0
786.7	51	781.9	47	796.6	45	791.6	49	770.3	2
779.6	2	776.9	2	785.9	3	782.9	3	761.7	66
725.4	37	710.2	32	722.1	37	717.1	30	687.7	16

C_6H_5CCH									
**M06-2X**		**B3LYP**		**CAM-B3LYP**		**wB97XD**		**MP2**	
ω	*I*	*ω*	*I*	*ω*	*I*	*ω*	*I*	*ω*	*I*
709.2	19	687.4	38	721.6	32	711.8	39	642.2	38
692.2	53	644.8	45	686.5	50	678.1	49	623.2	1
634.7	1	637.4	1	644.0	1	641.7	1	607.6	34
554.7	3	553.5	4	563.2	3	558.5	3	529.0	8
543.3	6	538.4	5	547.3	7	544.3	6	512.1	2
471.6	0	473.4	0	477.5	0	475.6	0	465.0	0
408.6	0	412.2	0	418.5	0	414.2	0	396.6	0
367.9	5	368.2	4	375.0	5	370.5	5	347.1	3
157.9	1	160.0	1	162.8	1	160.0	1	147.9	1
140.7	2	140.8	2	143.8	2	142.5	2	135.1	2

C_6D_5CCD									
**M06-2X**		**B3LYP**		**CAM-B3LYP**		**wB97XD**		**MP2**	
ω	*I*	*ω*	*I*	*ω*	*I*	*ω*	*I*	*ω*	*I*
2727.8	73	2700.5	77	2723.7	78	2721.5	73	2678.7	53
2396.3	3	2369.8	4	2388.2	3	2391.7	4	2397.0	4
2389.2	6	2363.1	10	2381.5	7	2384.4	10	2387.7	7
2381.1	3	2355.2	5	2373.4	4	2375.6	5	2379.1	2
2370.6	1	2343.6	2	2362.5	1	2365.0	1	2367.7	1
2360.8	0	2334.6	0	2352.6	0	2354.5	0	2358.9	0
2090.3	0	2057.8	0	2091.0	0	2084.4	0	2003.5	1
1639.4	2	1602.4	2	1644.5	2	1641.3	2	1604.7	4
1607.4	0	1568.9	0	1611.4	0	1608.9	0	1575.5	1
1422.6	9	1401.4	8	1429.6	9	1423.7	9	1456.0	0
1368.5	1	1354.7	1	1375.3	1	1369.1	1	1397.5	9
1299.6	1	1307.2	0	1305.2	1	1312.3	1	1335.2	0
1162.5	2	1155.2	2	1169.4	1	1164.0	2	1151.6	3
1052.8	0	1059.0	0	1067.3	0	1061.4	0	1042.2	0

C_6D_5CCD									
**M06-2X**		**B3LYP**		**CAM-B3LYP**		**wB97XD**		**MP2**	
ω	*I*	*ω*	*I*	*ω*	*I*	*ω*	*I*	*ω*	*I*
978.4	0	975.5	0	989.3	0	984.2	0	969.9	0
882.8	0	884.1	0	890.5	0	890.3	0	878.8	0
858.5	2	858.8	1	876.5	0	865.1	0	852.6	1
855.4	0	853.6	2	864.2	2	864.8	1	847.0	3
853.6	2	853.0	0	860.6	3	858.8	3	825.2	2
833.0	2	837.3	3	842.0	3	841.9	3	798.4	0
820.6	0	812.8	0	831.1	0	824.3	0	791.4	0
800.4	3	792.3	3	810.4	3	803.9	3	766.3	3
727.1	3	725.6	3	733.4	3	731.2	3	718.0	2
679.8	0	671.8	0	685.1	0	682.3	0	664.7	0
662.3	4	656.5	4	668.0	4	664.7	5	645.2	5
617.0	2	614.7	0	624.6	1	620.8	1	598.6	0
583.8	6	568.1	11	587.9	6	578.6	7	559.4	21
576.6	1	560.1	6	581.1	2	576.7	6	512.3	4
549.0	53	519.7	45	549.1	54	542.2	51	480.8	34
500.0	16	492.9	18	499.3	17	498.5	17	478.6	18
478.6	3	474.4	1	482.7	2	480.8	2	463.0	0
456.3	0	458.2	0	462.1	0	460.3	0	449.7	0
354.6	0	357.9	0	363.2	0	359.6	0	344.9	0
334.4	7	335.1	7	340.4	8	337.1	8	319.7	6
143.9	1	145.9	1	148.1	1	145.8	1	135.4	1
129.1	2	129.2	2	131.9	2	130.7	2	124.2	2

C_6H_5CCD									
**M06-2X**		**B3LYP**		**CAM-B3LYP**		**wB97XD**		**MP2**	
ω	*I*	*ω*	*I*	*ω*	*I*	*ω*	*I*	*ω*	*I*
3230.4	4	3196.9	4	3220.0	4	3224.1	5	3230.7	7
3225.6	9	3192.8	15	3215.5	11	3218.9	15	3223.5	12
3218.9	7	3185.4	13	3208.0	10	3211.1	12	3216.7	5
3210.3	2	3175.0	5	3198.3	3	3202.1	3	3207.7	1
3201.8	0	3166.3	0	3189.4	0	3192.8	0	3200.1	0
2727.8	73	2700.5	77	2723.7	78	2721.5	73	2678.7	53
2090.4	0	2057.8	0	2091.0	0	2084.5	0	2003.6	1
1672.9	2	1639.5	2	1679.9	2	1676.5	2	1637.2	4
1644.0	1	1609.7	1	1650.2	1	1647.3	1	1611.3	2
1534.1	14	1524.7	13	1547.0	15	1540.8	15	1511.4	13
1486.9	5	1477.3	4	1498.0	5	1491.7	5	1477.0	3
1349.8	0	1357.4	0	1366.7	0	1361.5	0	1443.3	1
1305.7	1	1311.6	0	1312.6	1	1317.9	1	1328.5	0
1226.9	1	1217.3	1	1232.8	1	1228.4	1	1217.6	2
1198.6	0	1200.5	0	1209.4	0	1209.9	0	1191.9	0
1175.8	0	1183.1	0	1185.7	0	1188.4	0	1173.3	0
1109.1	5	1102.0	5	1115.0	5	1113.4	5	1094.3	5
1060.9	4	1050.3	4	1065.8	4	1062.6	4	1047.0	5
1027.8	0	1019.0	0	1040.3	0	1032.6	0	1013.4	0
1021.6	0	1017.6	0	1033.9	0	1028.6	0	986.6	0
1007.0	0	997.8	0	1019.5	0	1011.8	0	973.2	0
955.7	3	945.3	3	966.3	4	959.7	3	920.1	3
873.4	0	863.6	0	880.6	0	877.0	0	854.5	0
786.4	47	781.5	43	796.4	41	791.3	45	766.0	1
775.5	2	772.9	2	781.9	2	778.9	2	760.9	60
708.7	31	710.1	34	721.5	36	717.0	34	687.3	19
638.3	0	639.0	0	647.3	0	644.3	0	623.5	0
591.6	6	566.5	4	595.4	6	585.2	6	529.0	7

C_6H_5CCD									
**M06-2X**		**B3LYP**		**CAM-B3LYP**		**wB97XD**		**MP2**	
ω	*I*	*ω*	*I*	*ω*	*I*	*ω*	*I*	*ω*	*I*
575.2	24	558.4	15	580.9	22	571.5	21	519.5	1
525.6	3	503.5	11	526.0	5	522.2	6	484.8	21
509.7	17	501.0	19	508.5	18	507.5	19	473.1	16
466.4	0	468.1	0	472.2	0	470.3	0	459.9	0
408.6	0	412.2	0	418.5	0	414.2	0	396.6	0
355.7	6	355.5	7	361.2	7	357.8	7	339.1	5
149.2	2	151.1	1	153.5	2	151.0	2	140.2	1
135.1	2	135.3	2	138.1	2	136.8	2	129.9	2

Table S6. Calculated vibrational transitions (ω, in cm^{-1}) and infrared intensities (I, in km mol^{-1}) of C_6H_5CC and C_6D_5CC radicals.

C_6H_5CC									
**M06-2X**		**B3LYP (C_s sym.)**		**CAM-B3LYP**		**wB97XD**		**MP2**	
ω	I	ω	I	ω	I	ω	I	ω	I
3235.4	5	3205.6	5	3227.9	4	3231.5	5	3257.3	2
3232.1	1	3203.3	3	3225.3	2	3228.2	4	3255.7	0
3223.1	2	3192.2	7	3214.8	4	3218.0	5	3244.8	0
3218.1	2	3184.1	4	3207.8	3	3211.9	3	3239.7	1
3206.6	0	3173.5	0	3196.3	0	3199.5	0	3225.2	0
2016.4	24	1906.1	110	2034.2	51	2018.8	48	2203.4	149
1635.1	111	1609.7	113	1640.1	94	1637.9	95	1787.4	257
1612.7	0	1577.3	1	1614.2	1	1613.1	0	1766.9	5
1505.3	6	1500.2	0	1518.3	2	1512.9	2	1560.2	5
1478.8	11	1467.0	10	1487.5	11	1481.8	11	1546.8	21
1347.7	7	1355.3	7	1361.6	6	1358.1	8	1360.7	2
1313.0	8	1311.7	4	1320.6	10	1323.0	8	1266.6	11
1220.6	31	1221.0	6	1232.7	28	1229.2	23	1255.9	16
1177.5	4	1182.8	2	1186.5	71	1189.0	4	1179.2	148
1172.9	101	1180.9	45	1185.9	4	1184.4	82	1176.0	0
1111.3	4	1103.3	5	1116.6	5	1115.1	5	1169.1	33
1051.3	0	1039.0	1	1054.6	1	1052.6	1	1165.3	0
1032.2	0	1025.6	0	1046.1	0	1038.8	0	1139.2	11
1016.5	0	1009.9	4	1028.3	0	1021.1	0	1128.8	4
1015.1	5	1006.9	0	1023.5	5	1019.5	5	1098.2	8
977.3	1	964.0	4	984.4	1	979.4	1	1024.2	9
867.7	0	855.6	0	872.0	0	868.8	0	994.7	0
790.0	28	791.1	30	802.0	24	796.6	27	907.8	55
783.6	0	777.2	12	790.5	0	787.5	0	820.4	7
681.7	41	684.9	41	697.2	44	691.6	44	765.4	44
625.6	0	625.4	0	633.3	0	631.7	0	668.6	0

C_6H_5CC									
**M06-2X**		**B3LYP (C_s sym.)**		**CAM-B3LYP**		**wB97XD**		**MP2**	
ω	*I*	*ω*	*I*	*ω*	*I*	*ω*	*I*	*ω*	*I*
509.9	1	500.7	1	512.0	1	509.4	1	648.3	1
482.7	20	488.9	47	489.1	17	485.9	18	497.5	1
472.2	5	474.8	1	479.1	3	476.9	3	491.3	5
385.8	0	387.9	0	395.1	0	390.8	0	440.9	2
278.6	23	262.3	87	246.2	24	256.8	24	434.8	0
145.0	4	145.9	4	148.5	5	147.1	4	148.4	2
121.7	11	114.2	22	103.6	20	113.6	16	141.5	4

C_6D_5CC									
**M06-2X**		**B3LYP (C_s sym.)**		**CAM-B3LYP**		**wB97XD**		**MP2**	
ω	*I*	*ω*	*I*	*ω*	*I*	*ω*	*I*	*ω*	*I*
2400.3	3	2376.2	4	2394.1	2	2397.33	3	2422.8	0
2394.8	2	2371.0	4	2389.0	2	2391.64	4	2420.0	1
2383.7	2	2360.3	5	2378.1	3	2380.28	4	2405.0	2
2376.2	1	2350.7	2	2369.5	1	2372.14	1	2397.3	0
2365.5	1	2340.9	0	2358.8	1	2360.64	1	2378.7	0
2016.3	24	1905.9	111	2034.1	50	2018.66	47	2203.2	150
1594.3	123	1566.0	124	1596.8	103	1594.96	105	1751.9	268
1566.5	0	1521.6	0	1563.6	0	1563.45	0	1730.1	9
1377.1	32	1363.1	6	1385.6	20	1380.38	22	1448.9	29
1366.5	10	1360.4	13	1371.8	10	1366.44	12	1445.7	10
1306.9	14	1303.0	4	1312.4	14	1316.96	12	1207.3	44
1156.1	77	1159.7	22	1171.2	56	1164.49	59	1162.2	104
1051.8	0	1057.0	0	1065.6	0	1059.94	0	1065.0	0
972.5	2	968.5	2	980.7	2	976.73	2	985.9	2
878.8	2	878.7	4	885.3	1	885.39	2	975.8	0
865.8	0	861.3	2	884.5	0	874.15	0	950.5	0
861.6	3	860.8	2	866.2	3	867.29	3	936.7	6

C_6D_5CC									
**M06-2X**		**B3LYP (*C_s* sym.)**		**CAM-B3LYP**		**wB97XD**		**MP2**	
ω	*I*	*ω*	*I*	*ω*	*I*	*ω*	*I*	*ω*	*I*
850.2	12	850.3	5	856.7	10	855.33	11	904.9	6
833.2	1	837.1	2	841.8	2	841.70	2	882.7	9
827.0	0	819.1	0	836.6	0	830.26	0	868.5	21
808.0	0	799.4	8	815.4	0	810.38	0	843.4	0
732.4	1	729.7	3	738.8	1	736.67	1	780.9	13
674.8	0	665.3	0	678.0	0	675.55	0	773.1	0
660.8	1	662.9	13	670.3	1	666.65	1	761.9	7
601.6	0	601.5	0	609.1	0	607.60	0	632.8	7
533.6	25	532.1	27	540.7	25	538.22	26	622.0	0
496.1	1	486.7	1	498.0	1	495.60	1	589.0	29
461.8	4	467.6	8	468.7	3	466.57	3	483.6	1
430.4	27	431.1	44	433.4	24	431.74	25	479.8	4
335.4	0	337.4	0	343.4	0	339.86	0	401.6	2
267.6	21	252.0	82	235.7	23	246.60	23	377.2	0
140.4	4	141.5	4	144.0	5	142.59	5	141.1	2
117.0	10	110.0	21	100.4	19	109.85	15	137.1	4

Table S7. Calculated vibrational transitions (ω, in cm^{-1}) and infrared intensities (I, in km mol^{-1}) of possible photolysis and annealing products at the M06-2X/aug-cc-pVTZ level.

**Ethynylphenyl radical C_6H_4CCH**					
**o-**		**m-**		**p-**	
ω	I	ω	I	ω	I
3484.4	93	3482.8	93	3222.0	0
3227.2	6	3227.6	3	3220.4	3
3215.6	5	3216.0	2	3206.2	0
3208.9	1	3214.0	1	3205.6	1
3199.3	0	3201.2	2	2728.0	74
2257.5	5	2252.2	5	2090.9	0
1665.0	0	1664.6	8	1638.3	0
1600.5	0	1582.5	5	1622.6	0
1490.1	13	1497.2	16	1495.2	14
1455.7	4	1446.2	5	1401.2	4
1313.3	0	1302.1	1	1311.4	1
1257.3	1	1285.8	1	1291.0	1
1228.9	2	1209.9	1	1218.0	1
1167.7	0	1176.0	0	1179.3	0
1130.4	4	1099.6	6	1107.2	4
1054.7	4	1068.8	5	1060.8	7
1013.5	0	1009.2	0	1002.1	0
997.4	1	1003.8	1	989.2	0
974.9	2	932.5	4	971.4	1
882.5	1	897.1	9	836.5	0
775.8	4	797.3	41	822.6	56
771.2	69	771.4	1	757.9	6
727.9	36	726.5	37	708.6	4
712.4	1	695.9	29	625.5	0
695.4	43	683.4	38	588.4	8

Ethynylphenyl radical C_6H_4CCH					
**o-**		**m-**		**p-**	
ω	*I*	*ω*	*I*	*ω*	*I*
630.1	2	626.6	0	566.0	28
553.5	0	566.1	0	514.9	0
527.2	4	544.3	8	509.8	17
464.8	1	464.9	0	469.8	1
419.8	2	421.0	2	398.1	0
365.7	3	362.9	4	370.4	15
155.1	2	158.7	1	149.5	1
138.7	3	139.4	2	140.6	1

Ethynylphenyl radical C_6D_4CCD					
**o-**		**m-**		**p-**	
ω	*I*	*ω*	*I*	*ω*	*I*
2730.9	68	3227.2	6	3482.8	93
2392.6	3	3215.6	5	3227.6	3
2380.1	3	3208.9	1	3216.0	2
2369.6	1	3199.3	0	3214.0	1
2358.6	0	2730.9	68	3201.2	2
2094.5	0	2094.5	0	2252.2	5
1643.1	0	1664.7	0	1664.6	8
1562.4	0	1600.4	0	1582.5	5
1402.0	9	1488.9	12	1497.2	16
1354.9	1	1455.6	5	1446.2	5
1276.9	1	1313.2	0	1302.1	1
1155.7	2	1255.7	1	1285.8	1
999.8	2	1224.5	2	1209.9	1
973.3	0	1167.5	0	1176.0	0

Ethynylphenyl radical C_6D_4CCD					
o-		m-		p-	
ω	*I*	*ω*	*I*	*ω*	*I*
872.2	0	1130.3	4	1099.6	6
850.7	2	1054.6	4	1068.8	5
843.1	0	1013.5	0	1009.2	0
830.2	2	997.3	1	1003.8	1
799.6	1	974.9	2	932.5	4
730.6	1	882.4	1	897.1	9
721.7	5	771.4	4	797.3	41
638.5	3	771.0	65	771.4	1
615.9	1	711.8	4	726.5	37
590.7	14	634.1	0	695.9	29
583.2	10	588.2	10	683.4	38
557.8	37	574.0	16	626.6	0
490.8	11	528.9	6	566.1	0
487.0	0	498.6	12	544.3	8
453.0	1	459.9	1	464.9	0
377.6	1	418.8	2	421.0	2
332.2	5	353.6	4	362.9	4
142.4	2	146.4	2	158.7	1
128.4	3	133.3	3	139.4	2

Ethynylphenyl radical					
C_6H_4CCD					
o-		m-		p-	
ω	*I*	*ω*	*I*	*ω*	*I*
3227.2	6	3482.8	93	2728.3	68
3215.6	5	3227.6	3	2391.4	2
3208.9	1	3216.0	2	2378.6	1
3199.3	0	3214.0	1	2375.0	0
2730.9	68	3201.2	2	2360.6	1
2094.5	0	2252.2	5	2090.3	1
1664.7	0	1664.6	8	1639.1	6
1600.4	0	1582.5	5	1555.2	4
1488.9	12	1497.2	16	1407.9	11
1455.6	5	1446.2	5	1356.1	2
1313.2	0	1302.1	1	1272.3	1
1255.7	1	1285.8	1	1152.5	1
1224.5	2	1209.9	1	1001.0	0
1167.5	0	1176.0	0	969.1	0
1130.3	4	1099.6	6	857.5	0
1054.6	4	1068.8	5	843.6	2
1013.5	0	1009.2	0	840.0	1
997.3	1	1003.8	1	831.9	6
974.9	2	932.5	4	787.2	4
882.4	1	897.1	9	740.2	1
771.4	4	797.3	41	740.0	1
771.0	65	771.4	1	645.2	3
711.8	4	726.5	37	614.2	5
634.1	0	695.9	29	584.6	3
588.2	10	683.4	38	569.8	9
574.0	16	626.6	0	539.7	41
528.9	6	566.1	0	515.3	3

Ethynylphenyl radical C_6H_4CCD					
o-		m-		p-	
ω	*I*	*ω*	*I*	*ω*	*I*
498.6	12	544.3	8	501.2	17
459.9	1	464.9	0	449.6	0
418.8	2	421.0	2	376.3	1
353.6	4	362.9	4	329.4	6
146.4	2	158.7	1	145.2	1
133.3	3	139.4	2	128.1	2

C_6H_4CC radical (triplet)					
o-		m-		p-	
ω	*I*	*ω*	*I*	*ω*	*I*
3232.0	3	3234.1	1	3228.4	1
3221.5	1	3221.0	3	3226.7	0
3216.9	0	3218.5	1	3212.5	0
3202.8	1	3204.6	0	3212.5	0
2022.1	40	2010.8	28	2024.9	45
1619.3	122	1626.7	77	1587.2	1
1576.3	1	1550.7	4	1568.5	144
1458.8	11	1469.5	10	1473.6	1
1432.5	10	1434.3	12	1402.7	9
1321.9	13	1300.5	9	1316.6	14
1239.5	2	1287.0	8	1300.2	0
1208.8	75	1184.0	40	1214.1	54
1160.3	31	1161.3	94	1160.8	62
1131.3	14	1103.1	10	1112.1	2
1043.3	1	1064.2	1	1044.3	0
1020.1	0	1018.4	0	999.3	0

C_6H_4CC radical (triplet)					
o-		m-		p-	
ω	*I*	*ω*	*I*	*ω*	*I*
991.2	4	997.6	4	991.6	0
990.0	0	957.5	1	991.5	4
882.3	0	904.4	3	826.5	0
780.7	0	811.0	25	823.1	42
774.4	46	776.0	0	763.7	3
700.5	16	664.6	38	716.2	10
619.4	2	617.8	0	606.9	2
490.0	10	509.3	1	509.5	1
489.8	0	503.1	7	494.2	32
462.8	5	464.4	3	475.2	7
403.3	5	393.9	5	372.7	0
276.1	23	266.9	27	293.6	13
143.2	3	147.0	4	144.2	5
120.1	8	119.7	13	126.3	13

C_6D_4CC radical (triplet)					
o-		m-		p-	
ω	*I*	*ω*	*I*	*ω*	*I*
2396.5	2	2396.7	1	2393.3	0
2384.8	1	2381.4	1	2390.0	0
2375.2	0	2379.3	0	2371.1	0
2362.2	2	2363.9	0	2368.8	0
2022.1	40	2010.7	28	2024.8	45
1589.1	130	1597.1	86	1561.3	2
1525.7	3	1515.4	3	1531.9	161
1360.1	26	1365.1	26	1357.2	16

C_6D_4CC radical (triplet)					
o-		m-		p-	
ω	*I*	*ω*	*I*	*ω*	*I*
1337.4	18	1352.8	25	1326.7	12
1273.2	15	1269.3	16	1310.9	6
1149.6	68	1142.0	77	1163.9	68
997.3	3	999.0	1	1006.1	0
966.6	4	965.3	0	965.9	1
871.0	1	854.6	2	861.5	0
851.9	3	847.1	0	850.4	13
849.6	0	844.9	8	837.0	0
829.2	5	832.4	12	819.1	2
806.6	0	791.2	0	814.2	0
728.0	1	750.5	0	712.3	4
727.9	0	746.2	0	688.2	8
637.7	1	656.6	1	642.0	0
599.5	1	598.7	0	628.7	5
578.3	23	528.4	32	589.9	2
479.8	0	496.3	1	494.7	1
456.1	5	470.8	7	468.4	6
439.9	13	453.9	2	432.7	38
359.8	8	349.2	7	323.6	0
265.9	21	256.6	25	287.0	11
139.8	4	143.1	5	140.1	5
116.4	8	115.4	12	123.5	13

C_6H_4CCXeH radical					
o-		m-		p-	
ω	*I*	*ω*	*I*	*ω*	*I*
3224.0	11	3226.2	5	3219.7	0
3213.2	8	3214.6	2	3217.9	7
3205.6	1	3213.0	3	3202.8	0
3196.3	0	3197.6	4	3202.3	3
2249.2	7	2244.0	6	2246.0	14
1757.8	1605	1766.3	1569	1750.0	1579
1660.6	25	1660.8	78	1634.6	0
1596.7	8	1577.1	11	1619.5	42
1487.2	50	1494.1	74	1494.9	39
1452.7	4	1443.9	7	1397.6	3
1310.9	1	1298.6	14	1308.4	0
1255.2	34	1281.0	6	1287.2	0
1229.4	54	1213.0	71	1229.2	95
1165.8	0	1173.2	0	1177.4	0
1126.8	4	1096.1	7	1104.9	5
1054.5	11	1067.9	13	1061.0	10
1011.0	0	1006.0	0	1001.2	2
996.0	4	1002.2	4	987.6	0
969.8	2	926.3	3	970.6	1
878.8	1	893.4	8	834.0	0
793.0	111	791.8	37	823.3	54
767.7	64	788.5	81	778.5	108
714.5	5	684.9	28	709.9	4
671.4	3	672.3	2	661.1	2
665.9	2	661.4	3	657.6	4
630.3	1	626.6	1	618.4	1
551.0	2	562.8	0	535.8	1
522.3	20	539.6	2	531.4	14

C_6H_4CCXeH radical					
o-		m-		p-	
ω	*I*	*ω*	*I*	*ω*	*I*
514.3	50	516.9	89	528.2	64
419.9	3	422.3	3	400.3	0
361.8	0	359.2	0	377.7	3
172.7	18	175.3	4	170.5	3
161.1	51	162.8	64	163.7	66
150.4	1	150.2	2	156.7	3
40.2	7	41.8	4	33.4	4
39.4	5	35.5	6	32.2	5

C_6D_4CCXeD radical					
o-		m-		p-	
ω	*I*	*ω*	*I*	*ω*	*I*
2390.2	6	2390	3.5	2385.0	0
2378.0	4	2377	0.6	2382.2	4
2366.8	1	2374	0.9	2363.0	2
2355.8	0	2358	2.6	2361.7	0
2249.1	5	2244	4.0	2245.9	12
1639.2	11	1636	34.7	1611.8	0
1558.5	2	1550	10.4	1590.8	13
1403.1	37	1410	48.2	1399.9	31
1354.0	0	1356	0.7	1321.4	0
1272.9	2	1267	16.3	1284.2	0
1248.3	761	1253	767.7	1243.7	711
1163.6	149	1161	131.3	1173.7	193
997.7	2	999	0.2	1005.6	0
972.2	2	968	0.9	975.1	0
873.7	11	856	2.5	867.7	8
848.8	2	844	3.6	856.0	0

C_6D_4CCXeD radical					
o-		m-		p-	
ω	*I*	*ω*	*I*	*ω*	*I*
842.3	0	840	0.8	832.2	1
829.5	2	831	5.4	816.4	3
796.7	1	786	3.6	806.0	0
739.7	101	758	79.3	726.6	100
730.3	1	736	0.4	700.4	14
636.6	3	641	2.9	649.0	0
609.8	1	607	2.3	610.6	2
588.1	23	545	12.6	601.9	1
524.0	2	536	0.2	530.5	0
508.3	65	531	20.0	520.7	64
508.2	12	506	90.3	498.4	12
484.0	1	488	1.9	480.7	4
479.9	3	488	4.2	463.4	7
378.8	4	378	3.2	356.5	7
336.7	0	334	0.4	346.8	0
166.8	31	167	5.5	161.9	3
156.9	35	160	59.2	161.3	63
143.3	1	142	1.7	150.8	3
38.7	7	40	3.8	31.9	3
37.8	5	34	5.1	31.2	5

3-Hexene-1-5-diyne (C_6H_4)				1,3,5-Hexatriyne (C_6H_2)	
E-		*Z*-			
ω	*I*	*ω*	*I*	*ω*	*I*
3482.1	0	3482.4	77	3479.5	0
3481.8	192	3481.6	74	3479.2	249
3200.9	7	3211.3	2	2382.7	0
3194.3	0	3195.4	5	2270.5	3
2254.7	3	2254.4	1	2163.9	0
2237.1	0	2239.2	2	1195.1	3
1694.1	0	1681.1	3	710.2	79
1327.0	0	1417.8	3	710.2	79
1289.9	1	1236.3	0	708.6	0
1043.4	14	1038.7	12	708.6	0
1033.1	0	1003.3	0	632.0	0
989.9	44	904.9	4	567.9	0
890.6	0	784.3	25	567.9	0
724.1	79	738.4	2	491.2	6
723.7	0	720.6	21	491.2	6
702.1	82	718.1	55	273.0	0
701.5	0	710.1	76	273.0	0
532.3	0	708.6	0	109.8	5
530.4	12	602.6	0	109.8	5
522.2	21	456.6	8		
357.7	0	355.5	28		
246.2	0	249.0	7		
127.1	1	221.1	0		
120.0	2	104.2	0		

C$_6$H$_6$CCH radical					
o-		m-		p-	
ω	*I*	*ω*	*I*	*ω*	*I*
3480.8	107	3483.3	87	3479.1	98
3230.4	7	3240.6	2	3217.2	1
3210.8	18	3212.7	11	3215.7	16
3205.1	0	3210.9	5	3196.6	3
3192.2	2	3195.4	3	3196.3	2
2993.6	5	2975.5	25	2992.8	6
2989.7	12	2972.6	7	2990.5	21
2195.9	22	2252.8	8	2160.2	12
1642.2	3	1627.0	1	1655.5	9
1532.5	2	1580.7	4	1564.9	0
1441.6	15	1444.9	1	1464.8	3
1429.8	2	1434.6	12	1440.1	17
1426.3	4	1391.6	1	1424.3	6
1331.4	1	1322.4	3	1358.6	1
1288.3	5	1296.2	7	1273.7	0
1205.7	0	1192.6	5	1259.5	2
1197.3	0	1192.5	0	1201.0	0
1188.1	1	1178.4	1	1181.8	3
1105.1	1	1093.2	1	1158.4	0
1016.6	5	1037.0	8	1001.7	0
1006.1	0	997.7	0	999.8	0
982.2	14	989.6	5	999.1	7
971.3	6	945.6	11	983.1	4
937.8	2	913.9	4	941.9	17
934.6	1	820.2	5	894.4	12
802.9	3	786.1	1	763.3	37
743.0	1	752.3	3	752.0	0
717.8	37	723.7	38	730.8	1

C_6H_6CCH radical					
o-		m-		p-	
ω	*I*	*ω*	*I*	*ω*	*I*
701.0	62	694.3	57	717.1	38
650.7	46	692.8	36	622.1	46
592.6	1	604.0	12	606.8	0
542.1	7	586.1	1	567.4	22
538.5	7	531.8	4	543.7	10
519.8	0	472.7	1	447.3	0
443.1	1	450.2	1	435.7	7
367.8	3	360.1	3	390.6	0
206.9	5	178.1	4	211.8	1
153.8	1	157.1	2	154.1	1
100.8	0	125.4	1	106.1	2

Phenylvinyl radical (C_6H_5CHCH)				alfa-Styryl radical	
E-		*Z*-		$C_6H_5CCH_2$	
ω	*I*	*ω*	*I*	*ω*	*I*
3268.8	1	3274.8	3	3230.4	6
3225.7	8	3224.9	10	3223.4	11
3216.2	17	3214.6	15	3220.2	3
3208.3	3	3205.6	2	3205.5	4
3198.9	0	3196.0	0	3200.3	2
3192.9	4	3191.7	5	3149.1	2
3123.0	5	3084.0	8	3087.8	19
1681.8	2	1686.6	1	1903.6	20
1669.7	3	1673.3	3	1610.4	2
1645.8	2	1646.6	2	1588.3	3
1537.5	11	1538.0	10	1493.4	14
1489.4	7	1490.0	7	1468.9	1

Phenylvinyl radical (C_6H_5CHCH)				alfa-Styryl radical	
E-		*Z-*		$C_6H_5CCH_2$	
ω	*I*	*ω*	*I*	*ω*	*I*
1356.5	1	1355.0	1	1435.3	1
1327.6	2	1326.5	2	1343.8	0
1256.8	0	1275.5	1	1295.0	1
1225.7	0	1224.4	1	1213.1	1
1201.8	0	1200.4	0	1164.3	0
1172.6	0	1172.5	0	1141.7	9
1112.5	7	1110.7	4	1109.3	4
1062.4	2	1062.0	3	1045.4	5
1021.8	0	1021.7	0	1012.0	0
1021.7	0	1021.6	0	999.6	0
1005.3	0	1007.3	0	986.8	4
957.2	16	956.8	5	963.5	0
902.1	47	900.5	9	915.8	42
898.9	36	888.3	1	911.0	3
870.7	1	870.8	0	841.9	0
791.9	3	790.5	73	771.2	2
788.1	11	766.0	5	762.6	49
722.2	79	702.8	12	688.9	34
678.2	0	659.2	20	627.3	0
631.2	0	631.7	0	554.5	3
548.9	1	552.8	14	476.0	7
466.3	4	463.1	24	458.1	0
437.4	1	451.3	0	447.8	0
409.3	0	410.2	0	389.5	0
235.5	0	224.7	7	211.3	0
214.0	0	223.5	3	148.9	1
37.9	0	83.5	0	75.8	3

Table S8. Calculated nucleus-independent chemical shifts (NICS, ppm) at the M06-2X level of theory.[a]

**Compound**	**$NICS(0)_{iso}$**	**$NICS(1)_{iso}$**	**$NICS(0)_{zz}$**	**$NICS(1)_{zz}$**
PhAc	-7.4	-9.8	-14.6	-29.0
C_6H_5CCXeH	-7.4	-9.8	-14.2	-28.7
Benzene	-7.1	-10.0	-16.3	-30.9

[a] Isotropic (iso) and out-of-plane (zz) NICS values were obtained by calculating the absolute NMR shielding using the GIAO method at the ring centers (0) and 1 angstrom above them (1). For comparison purposes, the calculated NICS values of benzene are also included. See references Foroutan-Nejad, C., Shahbazian, S. & Rashidi-Ranjbar, P. The electron density vs. NICS scan: a new approach to assess aromaticity in molecules with different ring sizes. *Phys. Chem. Chem. Phys.* **12,** 12630–12637 (2010) and Fallah-Bagher-Shaidaei, H., Wannere, C. S., Corminboeuf, C., Puchta, R. & Schleyer, P. V. R. Which NICS aromaticity index for planar π rings is best? *Org. Lett.* **8,** 863–866 (2006) for more details.
